# Genome-wide somatic mutation analysis of sinonasal adenocarcinoma with and without wood dust exposure

**DOI:** 10.1186/s41021-024-00306-8

**Published:** 2024-05-06

**Authors:** Lauri J. Sipilä, Riku Katainen, Mervi Aavikko, Janne Ravantti, Iikki Donner, Rainer Lehtonen, Ilmo Leivo, Henrik Wolff, Reetta Holmila, Kirsti Husgafvel-Pursiainen, Lauri A. Aaltonen

**Affiliations:** 1 Department of Medical and Clinical Genetics, University of Helsinki, Biomedicum Helsinki, Haartmaninkatu 8), PO Box 63, Helsinki, FI-00014 Finland; 2 Applied Tumor Genomics, Research Programs Unit, University of Helsinki, Biomedicum Helsinki, Haartmaninkatu 8), PO Box 63, Helsinki, FI-00014 Finland; 3 Finnish Cancer Registry, Unioninkatu 22, Helsinki, 00130 Finland; 4 Institute for Molecular Medicine Finland (FIMM), HiLIFE, University of Helsinki, Helsinki, Finland; 5 Molecular and Integrative Biosciences Research Programme, Faculty of Biological and Environmental Sciences, University of Helsinki, Helsinki, FI-00014 Finland; 6 Organismal and Evolutionary Biology Research Programme, Faculty of Biological and Environmental Sciences, University of Helsinki, Viikinkaari 9, Helsinki, 00014 Finland; 7 Institute of Biomedicine, Pathology, University of Turku, Kiinamyllynkatu 10, Turku, D 5035, 20520 Finland; 8 Turku University Central Hospital, Turku, 20521 Finland; 9 Finnish Institute of Occupational Health, PB 40, Helsinki, 00251 Finland; 10 Department of Pathology, University of Helsinki, PB 20, Helsinki, 00014 Finland; 11 Department of Biosciences and Nutrition, Karolinska Institutet, Huddinge, 141 83 Sweden; 12 iCAN Digital Precision Cancer Medicine Flagship, University of Helsinki, Helsinki, 00290 Finland

**Keywords:** Cancer, Sinonasal adenocarcinoma, Environmental exposure, Wood dust, Tobacco, Occupational health, Mutational signature, Formalin-fixed paraffin-embedded tissue

## Abstract

**Background:**

Sinonasal adenocarcinoma is a rare cancer, encompassing two different entities, the intestinal-type sinonasal adenocarcinoma (ITAC) and the non-intestinal-type sinonasal adenocarcinoma (non-ITAC). Occurrence of ITAC is strongly associated with exposure to hardwood dusts. In countries with predominant exposure to softwood dust the occurrence of sinonasal adenocarcinomas is lower and the relative amount of non-ITACs to ITACs is higher. The molecular mechanisms behind the tumorigenic effects of wood dust remain largely unknown.

**Methods:**

We carried out whole-genome sequencing of formalin-fixed paraffin-embedded (FFPE) samples of sinonasal adenocarcinomas from ten wood dust-exposed and six non-exposed individuals, with partial tobacco exposure data. Sequences were analyzed for the presence of mutational signatures matching COSMIC database signatures. Driver mutations and CN variant regions were characterized.

**Results:**

Mutation burden was higher in samples of wood dust-exposed patients (*p* = 0.016). Reactive oxygen species (ROS) damage-related mutational signatures were almost exclusively identified in ITAC subtype samples (*p* = 0.00055). Tobacco smoke mutational signatures were observed in samples of patients with tobacco exposure or missing information, but not in samples from non-exposed patients. A tetraploidy copy number (CN) signature was enriched in ITAC subtype (*p* = 0.042). CN variation included recurrent gains in COSMIC Cancer Gene Census genes *TERT*, *SDHA*, *RAC1*, *ETV1*, *PCM1*, and *MYC*. Pathogenic variants were observed most frequently in *TP53*, *NF1*, *CHD2*, *BRAF*, *APC*, and *LRP1B*. Driver mutations and copy number gains did not segregate by subtype.

**Conclusions:**

Our analysis identified distinct mutational characteristics in ITAC and non-ITAC. Mutational signature analysis may eventually become useful for documentation of occupation-related cancer, while the exact mechanisms behind wood dust-driven carcinogenesis remain elusive. The presence of homologous recombination deficiency signatures implies a novel opportunity for treatment, but further studies are needed.

**Supplementary Information:**

The online version contains supplementary material available at 10.1186/s41021-024-00306-8.

## Introduction

Sinonasal cancers are a rare group of malignant solid tumors originating in the nasal cavity and paranasal sinuses. In 2019, the incidence of sinonasal cancer in Finland was 1.6 per 100,000 person-years in men and 0.7 per 100,000 person-years in women [1]. They are often detected only at an advanced stage [2], and the location makes treatment difficult [3].

Sinonasal adenocarcinomas are divided into intestinal-type adenocarcinomas (ITAC) and non-intestinal-type adenocarcinomas (non-ITAC), with variable growth patterns and differentiation. Intestinal-type sinonasal adenocarcinoma (ITAC) is enriched in patients who have occupational exposure to wood dust, leather dust, formaldehyde, arsenic, nickel and chromium, and unspecific exposures related to textile manufacturing and construction industries [4]. Paint mists and organic solvents have been implicated as well [5, 6]. Sinonasal adenocarcinoma has a particularly strong association to wood dust exposure [5, 6].Histologically ITACs resemble malignant and normal forms of intestinal epithelium, and they can be distinguished immunohistochemically from non-ITACs by their expression of intestinal markers CDX2, CK20, and SATB2 [7]. ITAC is particularly strongly associated with exposure to hardwood dusts. In exposure to softwood dusts, occurrence of sinonasal adenocarcinomas is clearly lower, and the relative amount of non-ITACs to ITACs is higher.

Chronic inflammation has been posited as a likely driver of sinonasal carcinogenesis. When compared with sinonasal squamous cell carcinoma, sinonasal adenocarcinomas have on average a clearly higher COX-2 expression, as 12 out of 13 COX-2 expressing tumors were ITAC [8]. Inflammatory mechanisms are also supported by cultured cell lines expressing interleukins when exposed to different wood dusts [9]. A direct genotoxic effect of wood dust has been established [9, 10], with especially dust generated from composite wood products leading to acute DNA damage [10]. Used methods have measured biomarkers present only after short-term exposure, and thus the quantity and nature of damage accumulated over longer periods of exposure is unclear. Human bronchial epithelial cells can transform to a pre-cancerous phenotype in wood dust exposure in vitro, with DNA repair mechanisms malfunctioning in the transformed cells [11].

Mutational signatures derived from whole genome sequences of cancers can provide valuable clues to the etiopathogenesis of the disease [12]. To characterize mutation landscapes of ITACs and non-ITACs with and without exposure to wood dust we performed whole-genome sequencing (WGS) from archived formalin-fixed paraffin-embedded (FFPE) tumor DNA from 16 patients with documented wood dust exposure histories (10 exposed and 6 non-exposed).

## Materials and methods

### Sample set

We utilized archived Finnish sinonasal adenocarcinoma samples, a subset of a previously gathered and studied sample set from a multinational project concerning *TP53* mutation status in wood dust-related sinonasal cancer [13]. In this sample set, the individuals or their next-of-kin were contacted for background interviews concerning occupational exposure to wood dust and tobacco smoking habits, and wood dust exposure level and probability was subsequently evaluated by a panel of industrial hygienists based on industry, occupation, and period of employment of the individual. Whole genome sequencing was performed for FFPE tumors of 16 sinonasal adenocarcinoma patients (Table 1). We considered individuals with definite or probable wood dust exposure as exposed, and individuals with possible exposure as unexposed. Information on wood dust type and level of exposure was available in our study, but we did not consider these finer-resolution variables due to the small amount of sequenced samples and thus low statistical power of any subsequent analyses. Similarly, tobacco smoking was treated as a binary variable based on known smoking habits, without detailed consideration of exposure length, and those only exposed to second-hand smoking were classified as non-exposed. Samples were evaluated by HE stainings and immunohistochemistry (CK20 and CDX-2 stainings). Demonstration of intestinal differentiation was required for inclusion in the ITAC group, while lack of it led to inclusion in the non-ITAC group.

**Table 1:** Sample set and sequencing data summary

Sample	Wood dust	Exposure level	Exposure probability	Exposure type	Tobacco	Subtype	Tumor cell %	No. of libraries	Coverage (avg)	Coverage (sd)	Coverage (median)	Duplicate%
SNC12	exposed	medium	probable	S, H	NA	ITAC	50	1	30.3	69.2	29	13.8
SNC19	exposed	medium	definite	S	smoker	ITAC	20	1	34.3	69.9	33	16.3
SNC41	non-exposed	no exposure	no exposure	no exposure	non-smoker	ITAC	40	2	31	72.7	30	11.4
SNC48	non-exposed	no exposure	no exposure	no exposure	NA	non-ITAC	90	4	67.2	154.2	64	10.6
SNC72	non-exposed	no exposure	no exposure	no exposure	smoker	non-ITAC	25	2	33.8	96.2	32	11.3
SNC78	exposed	medium	probable	S, H	NA	ITAC	40	1	34.3	66	31	15
SNC105	exposed	medium	definite	S, H	smoker	ITAC	30	1	29.4	62.3	27	17.4
SNC131	exposed	low	definite	S, H	NA	non-ITAC	60	5	54.2	131.4	47	10.2
SNC142	exposed	medium	definite	S, H	NA	ITAC	30	3	82.9	164.1	79	10.2
SNC176	exposed	medium	definite	S, H	non-smoker	non-ITAC	80	1	36.5	66.4	33	14.6
SNC186	non-exposed	low	possible	no exposure	non-smoker	non-ITAC	90	4	53.6	121	48	10.1
SNC214	exposed	medium	definite	S, H	smoker	ITAC	70	5	84.4	196.7	78	7.5
SNC215	non-exposed	no exposure	no exposure	no exposure	smoker	non-ITAC	80	2	47.1	129.4	42	20.6
SNC229	exposed	medium	probable	S, H	smoker	ITAC	70	4	62.4	147.6	58	11.3
SNC232	non-exposed	no exposure	no exposure	no exposure	non-smoker	non-ITAC	80	1	28.6	63.5	27	12.4
SNC233	exposed	medium	definite	S	smoker	non-ITAC	70	1	30.7	72.6	29	11.9

Tumor cell percentage was determined by visual inspection of hematoxylin-eosin-stained tissue slides. Multiple libraries were prepared per sample when sample availability permitted. Coverage indicates the number of sample sequence reads aligning to reference sequence bases, presented as averages (avg), standard deviations (sd) and medians over the genome. Duplicate percentage indicates the fraction of sequence reads identified as duplicates by Picard’s MarkDuplicates tool, and is calculated as the average of all libraries when applicable. S = softwood exposure, H = hardwood exposure, ITAC = intestinal-type adenocarcinoma

### Sample preparation

The phenol-chloroform method was used to extract DNA from FFPE tissues. Different whole genome library preparation approaches were used to optimize the quality of sequence data. Libraries were prepared both in-house and by BGI Tech Solutions (PRC). All Illumina platform sequencing libraries were prepared using KAPA library amplification kits (Roche, CH), while BGISEQ-500 libraries were prepared at the sequencing service provider using the platform’s proprietary method. For KAPA libraries, either sonication using a Covaris sonicator (Covaris, Inc., USA) or S1 nuclease (Promega Corporation, USA) treatment was applied [14, 15]. NEBNext FFPE DNA Repair Mix (New England Biolabs, USA) was used in the preparation of a subset of KAPA libraries.

We initially ordered sequencing from BGI Tech Solutions using both Illumina X Ten (Illumina, USA) and BGISEQ-500 (BGI Tech Solutions, PRC). Sequencing libraries were produced by the service provider for this round. As the performance of these platforms has been estimated as more or less equal [16], and our summary quality control was in agreement, we decided to continue with Illumina technology as it allowed us control over library preparation.

In our first library preparation test round we followed standard KAPA HyperPrep kit library preparation protocol, with the exception of adding an enzymatic repair step using NEBNext FFPE DNA Repair Mix after DNA shearing with a Covaris sonicator (Covaris, Inc., USA), or replacing sonication with S1 nuclease (Promega, USA) treatment. These libraries were sequenced at Macrogen (Macrogen Europe BV, NL) with the NovaSeq 6000 platform (Illumina, USA), which was used for all remaining sequence data production in the project. The final library preparation protocol is presented in Additional file 1.

### WGS data processing

Overlapping raw sequence reads were error-corrected using BBMerge [17] from the BBTools suite version 37.62. Adapter sequences were removed with Trimmomatic version 0.39 [18]. Trimmed reads were aligned to human reference genome GRCh38 using BWA-MEM2 version 2.1 [19]. Read groups were added to aligned reads using the AddOrReplaceReadGroups tool from GATK version 4.1.9 [20]. When applicable, multiple libraries from the same sample were merged using SAMtools version 1.11 [21]. Duplicate reads were removed using MarkDuplicates from GATK. Remaining reads were sorted using SAMtools, after which GATK tools BaseRecalibrator and ApplyBQSR were applied. Our variant calling pipeline followed GATK best practices workflow for somatic short variant discovery, running the GATK release 4.0.4.0 tools SplitIntervals, M2, SumSubVcfs, MergeVCFs, SumFloats, MergeBamOuts, CollectSequencingArtifactMetrics, CollectF1R2Counts, LearnReadOrientationModel, CalculateContamination, Filter, FilterByOrientationBias, FilterAlignmentArtifacts, oncotate_m2, and FuncotateMaf [20]. We used an in-house panel of normals of 30 samples.

### Single base substitution-, indel-, and doublet signature analyses

Single nucleotide variants (SNVs) and indels passing all filters in GATK’s FilterMutectCalls tool were further filtered using BasePlayer [22], excluding calls with allelic depth less than 20, and less than three alternative allele reads. In the absence of paired normal tissue samples, we filtered germline variants by removing all variants with GnomAD v3 [23] all-population allele frequency over 0.00001, or variant allele fraction more than 0.35. Single base substitution (SBS), indel (ID), and doublet base substitution (DBS) mutational signatures were called using SigProfiler v1.1.4 [24]. SNV signatures were additionally extracted with a hierarchical Dirichlet process (HDP) mutational signature analysis method [25].

HDP *de novo* analysis was pursued as a means to extract novel signatures from the data, which would then be analyzed in detail. However, the observed components varied greatly in terms of credibility intervals of mutation categories comprising each component. This is due to the limited amount of samples which affected the amount of data available for inference. Furthermore, direct cosine similarity comparison using mean values and forgoing the use of credibility information in the presence of such variability is not robust. Thus, *de novo* signature analysis was considered only suggestive of HRD signature SBS3 (Additional file table S1), with emphasis placed on other methods during mutational signature results analysis.

Mutational signature data can be presented as scaled to each samples’ mutation count total, which presents relative contributions of different mutation processes within the tumor. We have chosen this approach due to large variability in mutation rates within the sample set, as relative importance of signatures were difficult to discern in samples with fewer total mutations. Clusterings and heatmaps may also be presented without such scaling. This affects the clustering and potentially visual interpretations as well, due to which both versions are presented in Additional file 2 figures S1–9.

### Copy number variant calls

First, we ran copy number variant (CNV) analysis with ASCAT [26], which failed to create CNV segments for the majority of samples due to low FFPE sample qualities. We were able to produce CNV segments with Control-FREEC v11.6 [27]. We used visualizations from ASCAT to adjust Control-FREEC “coefficientOfVariation” parameter and to validate the CNV calls (Additional file 2, Fig. S10). Resulting segments were merged if there were adjacent events with the same genotype and copy number. Due to low tumor cell percentage and non-existent allelic imbalance signals (Additional file 2, Fig. S10), we used the sample SNC72 as a control for all other samples.

### Copy number signature analysis

We used CNV segment results of Control-FREEC as an input to SigProfilerMatrixGenerator v1.2 [28] and then ran the resulting matrix through SigProfilerExtractor [24] v1.1.4 with default parameters to extract copy number (CN) signatures.

### Driver mutation analysis

We used ActiveDriver [29] to rank driver gene mutations in the sample set, with an input variant set consisting of all short variants passing all FilterMutectCalls filters, with an allele frequency of less than 0.0001 in the complete GnomAD set. To define the elements of interest for the ActiveDriverWGS analysis, all protein-coding sequence regions were extracted from Ensembl gene annotation release 104. A further analysis focusing on genes found in the COSMIC Cancer Gene Census (CGC) [30] was also produced. Mutation calls in sinonasal adenocarcinoma driver genes reported in previously published literature [31–34] were studied in detail; we excluded all GnomAD variants from unfiltered variant call files to remove germline variants, while minimizing the risk of not detecting mutations due to varying tumor cell percentages and intra-sample clonality. Remaining mutation calls were curated by inspecting read-level sequence data with BasePlayer [22] and finally filtering out variants predicted benign by the majority of tools in VarSome aggregated predictions [35]. All CGC genes were examined for doublet mutations matching the DBS2 signature. To compare *TP53* mutation calls produced by WGS and an earlier Sanger sequencing effort of the same sample set [13], all GnomAD variants were excluded from variants passing FilterMutectCalls filters and no further filtering was carried out. Significant genes in CNV peak regions were called by inserting the list of genes in each region to VarElect [36] and prioritizing genes present in CGC.

## Results

### Mutation burden is significantly higher in sinonasal adenocarcinomas with wood dust exposure

**Fig. 1:**
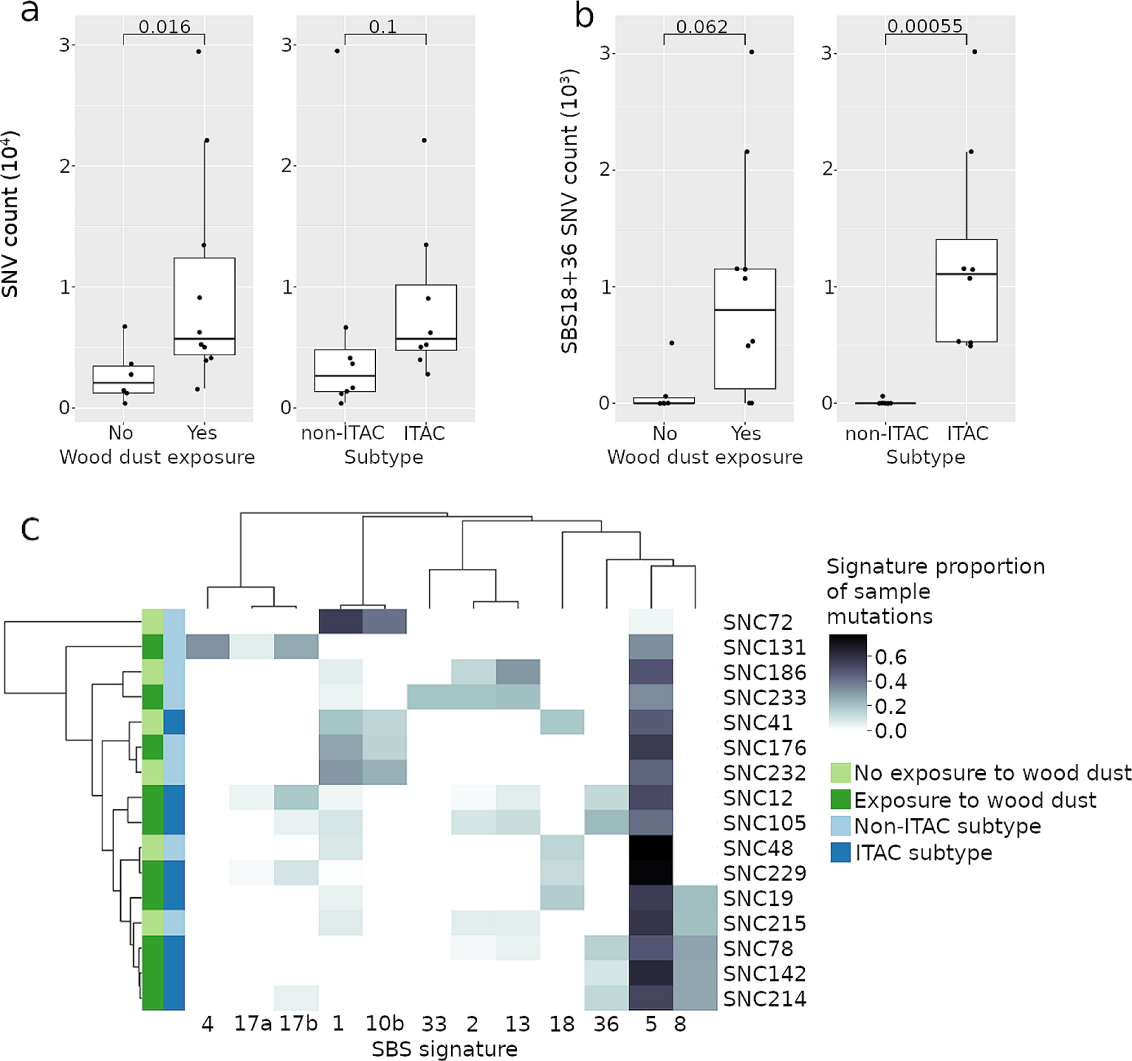
**(a)** Single nucleotide variant (SNV) counts of samples grouped by wood dust exposure and histological subtype. Sample difference tested with Wilcoxon rank-sum test. **(b)** Sums of mutations in signatures SBS18 and SBS36 grouped by wood dust exposure and histological subtype. Sample difference tested with Wilcoxon rank-sum test. **(c)** Single base substitution signatures of wood dust-exposed and non-exposed tumors produced with the SigProfiler method, with hierarchical clustering utilizing cosine distance and average linkage method. Signature activities are scaled as proportion of contribution to each sample’s total mutation count. An unscaled image is presented in Additional file 2 (Fig. S1)

We quantified SNV counts both overall and subsetted by assignment to specific mutational signatures, grouping the samples by wood dust exposure status and histological subtype (Fig. 1a and b). We found a significant trend (p-value = 0.016) of increased point mutation burden in the samples from patients with wood dust exposure (Fig. 1a). Mutation burden was not associated with histological subtype (*p* = 0.10, Wilcoxon rank-sum test Fig. 1a), and histological subtype was not associated with wood dust exposure status (*p* = 0.12, Fisher’s exact test). ITACs had lower tumor cell percentage estimates than non-ITACs (mean 43.8% vs. 71.9%, *p* = 0.023, Wilcoxon rank-sum test). Tumor cell percentage was not associated with wood dust exposure status (*p* = 0.15, Wilcoxon rank-sum test).

### SNV mutational signature profiling with SigProfiler identifies APOBEC signatures and ROS damage

We observed APOBEC SBS signatures SBS2 and SBS13 in the mutational signature analysis conducted with SigProfiler (Fig. 1c). Samples were concurrently positive for both signatures, and the signatures clustered together in hierarchical clustering. APOBEC signature positivity did not segregate with wood dust exposure or histological subtype. We observed *MUTYH* deficiency signature SBS36 in five wood dust-exposed samples, and a mutually exclusive reactive oxygen species (ROS) damage signature SBS18 with a very similar mutation spectrum, in two exposed and two non-exposed samples. Only one sample carried a potential loss-of-function mutation in *MUTYH*. Due to the high similarity of signatures SBS18 and SBS36 and the lack of somatic *MUTYH* mutations in the majority of samples, we compared these possibly ROS-induced signatures concurrently to detect any association with wood dust exposure or tumor subtype (Fig. 1b). We detected a statistically significant enrichment of ROS damage-type mutations in ITAC subtype tumors (*p* = 0.00055, Wilcoxon rank-sum test), and a borderline significant association with wood dust exposure (*p* = 0.062, Wilcoxon rank-sum test).

### Copy number, SNV, and indel mutational signature analyses identify homologous recombination deficiency as a potential novel feature in sinonasal adenocarcinoma

When sorted by cosine similarity, homologous recombination deficiency (HRD) signature SBS3 was among the best matches to *de novo* mutational process components extracted with HDP, with these novel components being present in the majority of samples (Additional file 2: Fig. S4 & Table S1). SBS8, a signature with HRD as a proposed etiology, was observed predominantly in wood dust-exposed samples in the SigProfiler analysis (Fig. 1c). Indel signature analysis with SigProfiler extracted among others ID6, an indel signature associated with HRD, which was observed in five samples (Additional file 2: Fig. S6).

CNVs appear as a frequent feature in beta allele frequency (BAF) segment plots of the sample set (Additional file 2: Fig. S10). CN signature analysis identified two signatures: tetraploidy signature CN2 and HRD signature CN17 (Table 2, Additional file 2: Figs. S8, S9). Neither CN signature was statistically significantly associated with wood dust exposure. CN2 was more common in ITAC subtype tumors (mean 12.4 variants vs. 2.3 variants, *p* = 0.042, Wilcoxon rank-sum test) and CN17 was more common in non-ITAC subtype (mean 15 variants vs. 6.4 variants, *p* = 0.07, Wilcoxon rank-sum test). HRD-associated SBS signature SBS8 was mostly present in samples positive for CN2 but not CN17 (4 out of 5 samples). Signature associations to tobacco exposure were not tested due to a large proportion of samples missing tobacco exposure data.

**Table 2:** Sample exposure status and CN variants assigned to CN signatures

Sample	Tobacco	Wood dust	Subtype	CN2	CN17
SNC142^a, b^	NA	exposed	ITAC	22	0
SNC12	NA	exposed	ITAC	0	16
SNC78^a^	NA	exposed	ITAC	19	0
SNC105	smoker	exposed	ITAC	19	0
SNC214^a^	smoker	exposed	ITAC	13	11
SNC229	smoker	exposed	ITAC	17	23
SNC19^a^	smoker	exposed	ITAC	9	0
SNC41	non-smoker	non-exposed	ITAC	0	1
SNC131^b^	NA	exposed	non-ITAC	0	24
SNC176	non-smoker	exposed	non-ITAC	0	21
SNC233^b^	smoker	exposed	non-ITAC	2	5
SNC48	NA	non-exposed	non-ITAC	0	3
SNC232^b^	non-smoker	non-exposed	non-ITAC	5	6
SNC186^b^	non-smoker	non-exposed	non-ITAC	9	14
SNC215^a^	smoker	non-exposed	non-ITAC	0	32

Table sorted by subtype. SNC72 was used as a control sample in CN calling and thus is not present in the result table. ITAC = intestinal-type adenocarcinoma. Columns CN2 and CN17 contain the count of variants matching COSMIC signatures for tetraploidy and homologous recombination deficiency, respectively. ^a^=sample positive for SBS8 in SigProfiler analysis. ^b^=sample positive for ID6 in SigProfiler analysis

### SNV, doublet, and indel tobacco exposure signatures are detectable from FFPE DNA

DBS signature analysis detected tobacco signature DBS2 in the sample set, with activity in samples with known tobacco exposure or missing data (Table 3). Based on signature presence in different analyses, at least three of the samples with missing tobacco smoke exposure data would be from smokers. Tobacco signature SBS4 was observed in one sample with SigProfiler (Fig. 1c), and in five samples with HDP. Samples that were positive for SBS signatures were also positive for DBS2, with the exception of SNC48, which had very few mutations overall, and a small proportion of total activity identified as SBS4 in the HDP analysis. Indel signature ID3, associated with tobacco smoking, was observed in two individuals with unknown smoking background and one known smoker. The known smoker was not positive for other tobacco-associated signatures. Practically no activity for any of the smoking signatures was detected in samples lacking tobacco exposure.

**Table 3:** Tobacco smoke exposure status and doublet base substitution count by DBS signature

Sample	Tobacco exposure	DBS2	DBS78B
SNC12	NA	10	0
SNC131^a, b,c^	NA	288	0
SNC142^b^	NA	49	86
SNC78^b, c^	NA	140	0
SNC48^b^	NA	0	15
SNC176	non-smoker	0	6
SNC186	non-smoker	0	32
SNC232	non-smoker	2	0
SNC41	non-smoker	1	7
SNC105	smoker	33	0
SNC19	smoker	34	0
SNC214^b^	smoker	196	0
SNC229	smoker	5	66
SNC233	smoker	0	10
SNC215^c^	smoker	0	24

SNC72 is missing from the DBS analysis as the sample did not contain any doublet mutations. DBS78B is a composite signature of DBS4, DBS6, DBS7, and DBS11. ^a^=SBS4 activity called in sample by SigProfiler. ^b^=SBS4 activity called in sample by HDP (Additional file 2: Fig. S2). ^c^=ID3 called in sample by SigProfiler

### Driver mutation analysis

No significantly enriched mutated genes were detected in the complete gene set analysis. When focusing the analysis on genes present in CGC, only *TP53* remained statistically significantly enriched after false discovery rate correction (*p* = 0.039, Chi-square tests and Benjamini-Hochberg-adjusted p-values calculated by ActiveDriverWGS).

In the comparison of results from Sanger sequencing and WGS, *TP53* mutation statuses were concordant in six out of eight samples when using positive Sanger sequencing results produced earlier [13] as reference (Table 4). Exact variant calls were reproduced in three samples, with one additional sample having a one-base position difference between WGS and Sanger variant calls. Two *TP53* Sanger-sequenced mutation-positive samples were not detected by WGS, while WGS detected one low allele fraction mutation in a sample observed as wild-type with Sanger sequencing. In one sample, WGS detected a second *TP53* mutation alongside a mutation detected with Sanger sequencing. Unspecified *TP53* exon mutations have been detected by capillary electrophoresis single strand conformation polymorphism (CE-SSCP) analysis in four samples [13], without successful subsequent validation by Sanger sequencing. The mutation status of these exons could not be validated by WGS, either.

**Table 4:** Comparison of *TP53* mutation calls from Sanger sequencing and WGS.

	Holmila et al. 2010			WGS		
sample	TP53 genotype	exons	change	TP53 genotype	exons	change
SNC12	mutated	5*, 7	Gly244Cys	Het(3/22)	7	Arg248Gln
SNC19	WT	-	-	WT	-	-
SNC41	mutated	6*, 7*	-	WT	-	-
SNC48	WT	-	-	WT	-	-
SNC72	WT	-	-	Het(3/30)	8	Arg282Gln
SNC78	mutated	7	Arg249Gly	Het(7/15)	7	Gly244Asp
SNC105	WT	-	-	WT	-	-
SNC131	mutated	5	Asp184Ala	WT	-	-
SNC142	mutated	5	Tyr163Cys	Het(10/70), Het(25/55)	5, 7	Tyr163Cys, Asn235Ser
SNC176	WT	-	-	WT	-	-
SNC186	mutated	7	Cys242Gly	Het(13/26)	7	Cys242Phe
SNC214	mutated	5	Thr155Pro	Het(34/66)	5	Thr155Pro
SNC215	WT	-	-	WT	-	-
SNC229	mutated	8*	-	WT	-	-
SNC232	mutated	5*, 7	Gly245Val	WT	-	-
SNC233	mutated	5	Cys135Tyr	Het(5/22)	5	Cys135Tyr

*TP53* genotype presented as status in capillary electrophoresis single strand conformation polymorphism (CE-SSCP) and Sanger sequencing results from Holmila et al. 2010 and genotype calls with relative depth of alternative allele sequence reads in WGS. ∗=exon mutation detected only in CE-SSCP, with exact mutation unknown

**Fig. 2:**
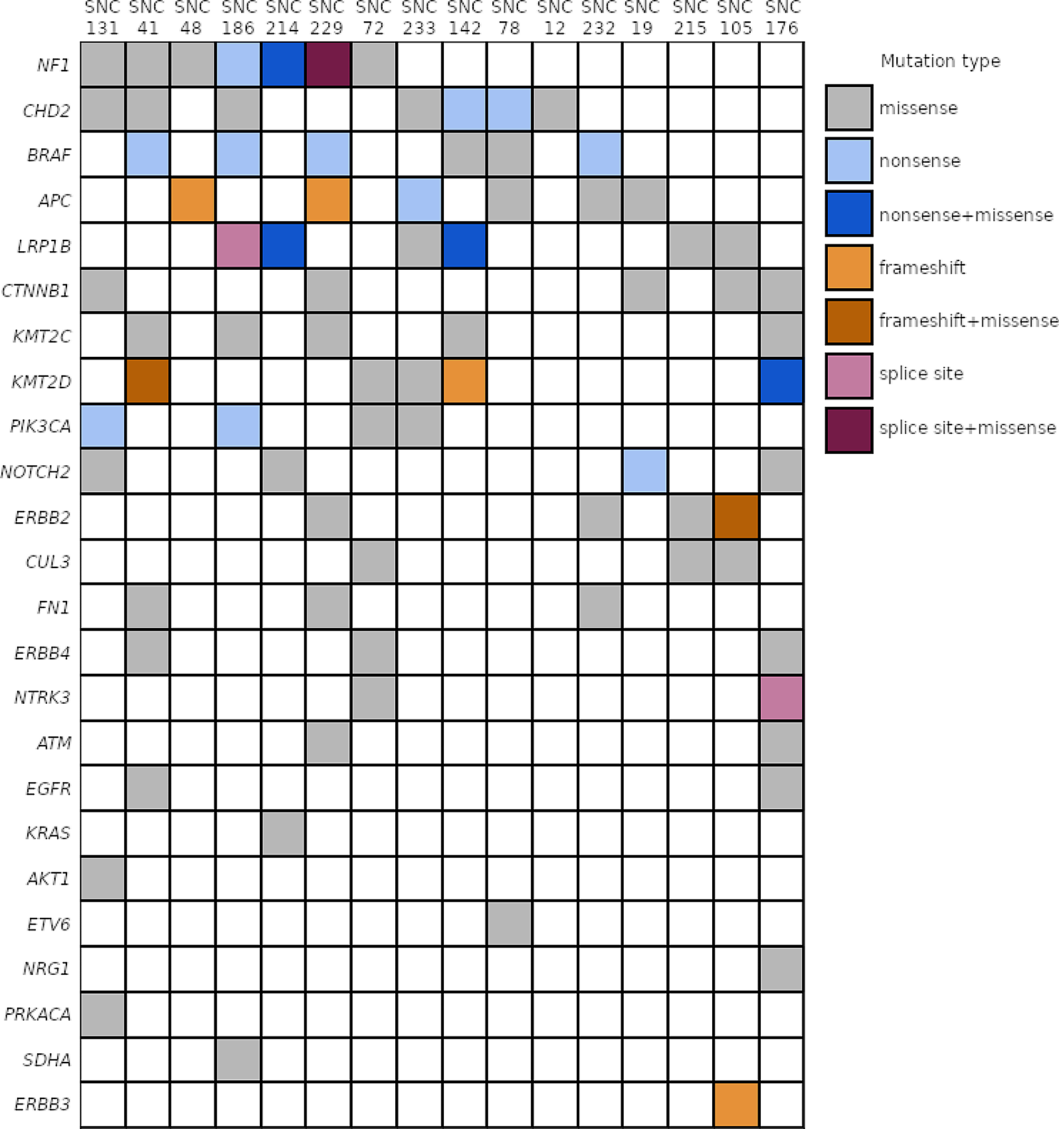
Mutation status of previously reported sinonasal adenocarcinoma driver genes. Columns represent samples, and rows represent genes. Color indicates type of mutation, with darker hue signifying the presence of additional missense mutation calls in the same gene, in the same sample.

Analyzing mutation status of previously reported sinonasal adenocarcinoma driver genes revealed no significant association of mutations with either ITAC subtype or wood dust exposure. The most significant enrichment was for *PIK3CA* mutations in non-ITAC subtype (four mutations in non-ITAC tumors and zero mutations in ITAC tumors, *p* = 0.077, Fisher’s exact test) and *CTNNB1* mutations by wood dust-exposure (five wood dust-exposed samples positive and zero non-exposed, *p* = 0.093, Fisher’s exact test). The most often mutated driver genes were *NF1* and *CHD2* (Fig. 2). We observed two *BRAF* nonsense mutations Arg239Stop and Arg558Stop in our data, each in two samples, and a *KRAS* Gly12Ala substitution mutation. *EGFR* was mutated in two samples, causing Gly63Arg and Val308Ile substitutions, while Leu858Arg was not observed. Nonsynonymous *BRCA1* and *BRCA2* mutations were detected, but none of these were predicted to be pathogenic.

Copy number analysis revealed copy number gain peaks at 5p15.3 (11 out of 16 samples), 7p21.3-p22.1 (11 samples), 8p21.3-p22 (10 samples), and 8q24.13 (8 samples). A loss peak was observed at 5q14.3-q15 (6 samples). The gain peaks overlap *TERT* and *SDHA* in chromosome 5, *RAC1* and *ETV1* in chromosome 7, and *PCM1* in chromosome 8p with another peak near *MYC* in 8q. *EGFR* amplification was observed in ITAC samples SNC78, SNC105, SNC214, and SNC229, with no observations in non-ITAC samples (*p* = 0.077, Fisher’s exact test). Copy number variants did not associate with histological subtype or wood dust exposure history. Figures of copy number variation and a table of sample-specific results for the most commonly affected genes is available in Additional file 2 (Fig. S11, Table S2).

Analyzing the occurrence of doublet mutations matching the DBS2 signature mutation spectrum in genes present in CGC, we observed a CC > AA mutation at 2:47836001–47,836,002, causing a splice site mutation and replacing Trp196 with cysteine in the gene *FBXO11*, and a GG > TT mutation at 3:30674125–30,674,126, causing replacements Met425Ile and Ala426Ser in the tumor suppressor gene *TGFBR2*. These doublet mutations occurred in SNC105 and SNC19, respectively, both samples being from smokers.

## Discussion

We have performed whole-genome sequencing for 16 archival FFPE samples of sinonasal adenocarcinoma to study any possible mutational signatures and driver genes shared by the sample set, and the association between mutational landscape and environmental exposures. Although the sample material was challenging, as archival FFPE tumor tissues were utilized instead of fresh tissue samples, we were able to extract novel characteristics for this tumor type. We used multiple different approaches to strengthen the confidence of our findings.

There were several limitations to this study, the most definitive of which was the small sample size. Lack of statistical power impeded determination of association between mutational characteristics and exposure information, especially in tobacco smoke exposure where data was incomplete. In driver gene enrichment analysis, systematically testing every gene for mutation enrichment left no genome-wide significant associations after adjusting for multiple comparisons. Both ITACs and non-ITACs can be subdivided into more defined subtypes, but due to the small number of cases in our analysis, we have preferred to carry out the analysis using this main distinction. The second issue was low tumor cell percentage in some samples. This limited our ability to detect SNVs in areas of low coverage, and thus we may not have detected some significant driver mutations. Our third issue was the use of FFPE material; aged paraffin blocks have accumulated damage and this caused noise in the variant analysis. The effects of FFPE damage were mitigated with sequencing library preparation method choices, multiple library sequencing, and maximizing sequencing coverage. While we assume sequencing artifacts to be diluted by true positive signals in mutation signature analysis, we have focused our driver mutation calling efforts to a limited subset of genes, allowing manual curation of the WGS mutation calls.

Due to limited resources and high variability in the availability of healthy tissue for sequencing, we did not have germline sequence data for the individuals. We removed germline variants with two procedures: by removing all variants present in the GnomAD dataset, and with a heuristic method based on variant allele fractions. As tumor cells amount to only a minority of the cells within the samples, allelic fractions of somatic heterozygous variants of tumor cells are lower in the sequencing library than those of germline variants, unless copy number events have also occurred in the region. The threshold value of 0.35 was chosen by inspecting the variant allele fraction distribution of each sample.

Strand-split artifact reads (SSARs) have been described as a feature of FFPE high throughput sequencing data, being almost completely absent from sequence data generated from fresh-frozen tissue DNA [15]. SSARs are chimeric, non-contiguous reads thought to arise from single-stranded DNA annealing together. By definition SSARs have a supplementary alignment occurring on the opposite strand within 500 base pairs of the primary alignment. Using S1 endonuclease during DNA extraction has been shown to improve sequencing library quality [14]. S1 has also been observed to mitigate the negative quality effects of SSARs, when used as a pretreatment to a conventional sonic shearing workflow [15]. In the initial stages of our study, we evaluated the effects of S1 endonuclease treatment in pre-extracted DNA, with additional preprocessing with an enzymatic repair mix in some tests. Libraries produced with S1 treatment were not sonicated, as FFPE DNA is already severely fragmented, and sequencing libraries are subjected to fragment size selection during production. Due to the small amount of samples, and the small size of individual samples, the amount of material placed severe limitations to our optimization efforts. Data from the first test round suggested that either applying an enzymatic repair step, or an S1 nuclease treatment without sonication, modestly improved oxidative artifact error rates as calculated by Picard’s CollectOxoGMetrics tool (Additional file 2: Fig. S12). Alignment-related metrics appeared quite similar between the different treatments, with the level of variability within this subset also being possible due to batch effects (Additional file 2: Table S3). The second tested method consisted of incubating sample DNA with an enzymatic repair mix prior to S1 treatment. This method proved to be effective in removing SSARs (Additional file 2: Fig. S15) and was chosen as the final library preparation protocol for this study. However, due to the limited amount of libraries produced with S1 treatment without enzymatic repair, it is difficult to determine whether the repair step is needed, and if equal results would be achieved with S1 alone. A comparison of all sequencing libraries is provided in Additional file 2 Table S3. SSAR artifact reduction when the protocol is applied is shown in Additional file 2 Fig. S16, and key alignment metrics in Additional file 2 Table S4.

Analysis of samples from wood dust exposed individuals and cell lines have demonstrated the presence of short-term damage following exposure, with in vivo long-term effects remaining less known due to limitations of the employed assays [10]. Furthermore, while cell line results indicate mutagenic potential in a range of different wood species [9], only exposure to dust from composite wood products as compared to natural wood caused a statistically significant difference in DNA damage in workers [10]. Here, we have observed a statistically significant increase in total mutation burden in sinonasal adenocarcinomas from wood dust-exposed individuals, indicating that short-term mutagenic effects of wood dust extrapolate to the accumulation of mutations over a longer time period. Wood dust type was not considered in our analysis.

While epidemiological studies have consistently associated wood dust exposure with risk for sinonasal adenocarcinoma, pathological studies associate ITAC specifically with hardwood dust exposure [37]. The significant association of ITAC subtype and sums of ROS damage-associated SBS signatures 18 and 36 is in agreement with previous information of COX-2 expression being a feature of ITACs [8]. COX-2 expression is known to be induced by ROS [38], and ROS generation by wood dust has been described [39]. Thus our results support the idea that persistent inflammation caused by wood dust drives specifically ITAC subtype development. Even though *MUTYH* deficiency has been proposed to play a role in the etiology of SBS36, we detected only one such mutation in our dataset, and thus it is possible that the accumulation of signature-matching oxidative damage was driven by some other mechanism than deficiencies in base excision repair.

APOBEC mutations are associated with inflammation-related interferon-gamma gene expression signature in head and neck squamous cell carcinoma [40]. Here, we did not observe any difference in APOBEC signature mutation count by either wood dust exposure or histological subtype, and only partial overlap with ROS signatures. SBS10b, a signature caused by polymerase epsilon (*POLE*) exonuclease domain mutations, observed in a subset of the samples, was possibly a false positive signal caused by FFPE artifacts as only one sample with activity in this signature carried a nonsynonymous *POLE* mutation, which was predicted functionally benign. Furthermore, the SBS10b signature consists predominantly of C > T mutations which are a common artifact in FFPE-derived DNA. SBS2 is similarly a signature characterized by C > T mutations, and was mutually exclusive with SBS10b, suggesting that the activity of these two signatures may be generated by sequencing artifacts caused by FFPE damage. However, APOBEC signature SBS13 was concurrently observed with SBS2; SBS13 is characterized by a dissimilar mutation profile to SBS2, and was not observed in samples only positive for SBS10b, which increases our confidence in this finding. Mismatch repair (MMR) pathway genes have been found to remain normally expressed in ITACs [41], with a single sample in a set of 41 ITACs displaying microsatellite instability [42]; our results agree with this as we did not observe any significant activity in mutational signatures associated with defective DNA mismatch repair, nor did we observe mutations in MMR genes.

Previous studies have characterized chromosomal abnormalities in both sinonasal adenocarcinoma in general [43], and focusing only on ITAC [44]. The peaks observed in our CN analysis are largely in agreement with these publications, which lends credibility to our results. While the gain peak at chromosome 8q in our data did not occur at proto-oncogene *MYC*, the peak hit approximately 550 kilobases to 5 megabases upstream of the gene, covering known *MYC* enhancers [45]. In 7 out of 8 samples the CN gain covered the gene as well. The occurrence of *MYC* CN gain in sinonasal adenocarcinoma has been discussed before [43, 44] and our results add to the topic of *MYC* overexpression being a significant feature in this cancer. *TERT* amplification, to our knowledge, has not been discussed in the context of sinonasal adenocarcinoma. While the loss peak in chromosome 5 contained no CGC genes, RAS suppressor gene *RASA1* is a plausible candidate gene in this region, as its inactivation promotes RAS signaling activation and subsequent tumorigenesis [46].

The apparent segregation of CN signatures CN2 and CN17 by histological subtype is surprising, especially as HRD-related SBS, ID and CN signature activity does not overlap well, for example samples positive for SBS8 had limited CN17 activity at best. HRD signature SBS3 has been previously observed in whole exome sequence data of a patient-derived ITAC cell line [47]. We did not observe similar mutations, however observation of these signatures warrants future study into the subject. Validation of this finding as a biological feature in sinonasal adenocarcinoma would open new prospects for its treatment, as poly-ADP ribose polymerase inhibitor (PARPi) treatment is effective in homologous recombination deficient breast and ovarian cancer [48].

The presence of tobacco signatures in samples from smokers, but not in those of non-smokers, lend additional credibility to the notion that significant environmental exposures can be detected in technically challenging FFPE samples. The carcinogenic role of tobacco smoke is supported by our observation of DBS2-matching doublet mutations occurring in tumor suppressor genes *FBXO11* and *TGFBR2* in the samples of two individuals with a smoking history.

Our SNV and indel analysis focused on already established driver genes. Interestingly, we did not observe enrichment of any driver gene by subtype, possibly due to the limited size of the sample set.

*TP53* was the only CGC gene to remain statistically significantly enriched after correction for multiple testing, being mutated in 7 out of 16 samples (44%). *TP53* variant calls produced from WGS data were partially discordant with Sanger sequencing results produced from the same samples a decade earlier, possibly due to accumulation of further damage over time, or due to differences in sample processing. When considering only the ITAC subtype, 4 out of 8 samples (50%) had a mutation. This is in agreement with values presented elsewhere, as studies focusing on the ITAC histology have reported *TP53* mutations in 58% [34] and 41% [49] of samples, and non-functional p53 in 42% of samples [50].

Approximately half of ITACs have *EGFR* copy number gains resulting from either chromosome 7 polysomy or focal gene amplification, with *EGFR* overexpression occurring in some 7–21% of samples [51, 52]. Overexpression is also observed in the absence of copy number variation [52]. In our sample set, we observed *EGFR* amplification in four out of eight ITACs studied. Our finding of truncating *BRAF* mutations in four samples is surprising, as oncogenic *BRAF* mutations predominantly increase the kinase activity, the most significant mutation by far being substitution of *BRAF*’s 600th amino acid valine to a glutamic acid (V600E). The truncating *BRAF* mutations in this sample set have been previously observed in the context of other cancers [53–55]. We also observed missense *BRAF* mutations in some samples, however none of these were V600E or predicted pathogenic. *BRAF* V600E appears to be rare in sinonasal adenocarcinoma, as previous studies characterizing this mutation in ITACs have reported zero observations in 57 samples [52] and a single observation in 34 samples (2.9%) [51]. *KRAS* mutations have been observed in 5.9% [51] and 12% of ITACs [52], and in 13% of a sample set consisting of both non-ITACs and ITACs [56], and thus encountering a single *KRAS* mutation in this study matches the reported rates of observation.

In conclusion, our results support that FFPE, a challenging but underutilized source material in medical genetic research, can be used in whole-genome studies of environmental exposure with certain precautions. The long-term mutagenic effect of wood dust exposure is demonstrated by the observations of increased mutation burden in exposed individuals and ROS mutational signatures in ITAC subtype tumors. We were not able to connect total mutation burden with any specific mutational signature or driver gene mutation. We have detected HRD signatures as a common feature in this tumor type, which has potential clinical significance. Inconsistency between different types of HRD mutational signatures, combined with our small sample size, underlines that further studies are needed to validate this finding. Single base, doublet, and indel tobacco exposure signatures were observed in known smokers or those with missing data, but not in non-smokers.

### Electronic supplementary material

Below is the link to the electronic supplementary material.

Additional file 1. Library preparation protocol. This additional file contains the final library protocol that was used to produce most of the libraries after the initial library production and sequencing tests.

Additional file 2. Supplementary figures and tables. This additional file contains mutation signature plots with hierarchical clusterings, BAF segment graphs, copy number call graphs and tables, quality control figures and tables, and supplementary alignment distance plots that visualize the effect of sequencing library preparation protocol on the number of chimeric reads.

## Data Availability

Raw sequence data generated in this study cannot be shared due to restrictions in the permissions given for this investigation and the current legislation in Finland. Such permissions can be applied from Findata, the Social and Health Data Permit Authority, and pending successful application the raw data will be made available.

## References

[1] Pitkäniemi J, Malila N, Tanskanen T, Degerlund H, Heikkinen S, Seppä K. Cancer in Finland 2019. Helsinki; 2021.

[2] Koivunen P, Mäkitie AA, Bäck L, Pukkila M, Laranne J, Kinnunen I (2012). A national series of 244 sinonasal cancers in Finland in 1990–2004. Eur Arch Otorhinolaryngol.

[3] d’Errico A, Pasian S, Baratti A, Zanelli R, Alfonzo S, Gilardi L (2009). A case-control study on occupational risk factors for sino-nasal cancer. Occup Environ Med.

[4] Binazzi A, Ferrante P, Marinaccio A (2015). Occupational exposure and sinonasal cancer: a systematic review and meta-analysis. BMC Cancer.

[5] d’Errico A, Zajacova J, Cacciatore A, Alfonzo S, Beatrice F, Ricceri F (2021). Exposure to occupational hazards and risk of sinonasal epithelial cancer: results from an extended Italian case–control study. Occup Environ Med.

[6] IARC Working Group on the Evaluation of Carcinogenic Risks to Humans. Arsenic, metals, fibres, and dusts. In: IARC monographs on the evaluation of carcinogenic risks to humans. 2012. 100(PT C): 11. PMC4781271 23189751

[7] Skalova A, Sar A, Laco J, Metelkova A, Miesbauerova M, Steiner P (2018). The role of SATB2 as a diagnostic marker of Sinonasal Intestinal-type Adenocarcinoma. Appl Immunohistochem Mol Morphol.

[8] Holmila R, Cyr D, Luce D, Heikkilä P, Dictor M, Steiniche T (2008). COX-2 and p53 in human sinonasal cancer: COX-2 expression is associated with adenocarcinoma histology and wood-dust exposure. Int J Cancer.

[9] Bornholdt J, Saber AT, Sharma AK, Savolainen K, Vogel U, Wallin H (2007). Inflammatory response and genotoxicity of seven wood dusts in the human epithelial cell line A549. Mutat Res Toxicol Environ Mutagen.

[10] Bruschweiler ED, Wild P, Huynh CK, Savova-Bianchi D, Danuser B, Hopf NB. DNA damage among Wood Workers Assessed with the Comet Assay. Environ Health Insights. 2016;10:EHI.S38344. 10.4137/EHI.S38344 PMC4927022 27398027

[11] Staffolani S, Manzella N, Strafella E, Nocchi L, Bracci M, Ciarapica V (2015). Wood dust exposure induces cell transformation through EGFR-mediated OGG1 inhibition. Mutagenesis.

[12] Alexandrov LB, Nik-Zainal S, Wedge DC, Australian Pancreatic Cancer Genome Initiative, ICGC Breast Cancer Consortium, ICGC MMML-Seq Consortium (2013). Signatures of mutational processes in human cancer. Nature.

[13] Holmila R, Bornholdt J, Suitiala T, Cyr D, Dictor M, Steiniche T (2010). Profile of TP53 gene mutations in sinonasal cancer. Mutat Res Mol Mech Mutagen.

[14] Chun S-M, Sung CO, Jeon H, Kim T-I, Lee J-Y, Park H (2018). Next-generation sequencing using S1 nuclease for poor-quality Formalin-Fixed, paraffin-embedded tumor specimens. J Mol Diagn.

[15] Haile S, Corbett RD, Bilobram S, Bye MH, Kirk H, Pandoh P (2019). Sources of erroneous sequences and artifact chimeric reads in next generation sequencing of genomic DNA from formalin-fixed paraffin-embedded samples. Nucleic Acids Res.

[16] Patch A-M, Nones K, Kazakoff SH, Newell F, Wood S, Leonard C et al. Germline and somatic variant identification using BGISEQ-500 and HiSeq X Ten whole genome sequencing. PLOS ONE. 2018;13:e0190264. 10.1371/journal.pone.0190264 PMC5761881 29320538

[17] Bushnell B, Rood J, Singer E. BBMerge – Accurate paired shotgun read merging via overlap. PLOS ONE. 2017;12:e0185056. 10.1371/journal.pone.0185056 PMC5657622 29073143

[18] Bolger AM, Lohse M, Usadel B (2014). Trimmomatic: a flexible trimmer for Illumina sequence data. Bioinformatics.

[19] Vasimuddin Md, Misra S, Li H, Aluru S. Efficient Architecture-Aware Acceleration of BWA-MEM for Multicore Systems. 2019 IEEE Int Parallel Distrib Process Symp IPDPS. Rio de Janeiro, Brazil: IEEE; 2019. p. 314–24.

[20] de Auwera GAV, O’Connor BD. Genomics in the cloud: using Docker, GATK, and WDL in Terra. First edition. Beijing Boston Farnham Sebastopol Tokyo: O’Reilly; 2020.

[21] Li H, Handsaker B, Wysoker A, Fennell T, Ruan J, Homer N (2009). The sequence Alignment/Map format and SAMtools. Bioinformatics.

[22] Katainen R, Donner I, Cajuso T, Kaasinen E, Palin K, Mäkinen V (2018). Discovery of potential causative mutations in human coding and noncoding genome with the interactive software BasePlayer. Nat Protoc.

[23] Karczewski KJ, Francioli LC, Tiao G, Cummings BB, Alföldi J, Wang Q (2020). The mutational constraint spectrum quantified from variation in 141,456 humans. Nature.

[24] Islam SMA, Díaz-Gay M, Wu Y, Barnes M, Vangara R, Bergstrom EN (2022). Uncovering novel mutational signatures by de novo extraction with SigProfilerExtractor. Cell Genomics.

[25] Li Y, Roberts ND, Wala JA, Shapira O, Schumacher SE, Kumar K (2020). Patterns of somatic structural variation in human cancer genomes. Nature.

[26] Van Loo P, Nordgard SH, Lingjærde OC, Russnes HG, Rye IH, Sun W (2010). Allele-specific copy number analysis of tumors. Proc Natl Acad Sci.

[27] Boeva V, Popova T, Bleakley K, Chiche P, Cappo J, Schleiermacher G (2012). Control-FREEC: a tool for assessing copy number and allelic content using next-generation sequencing data. Bioinformatics.

[28] Khandekar A, Vangara R, Barnes M, Díaz-Gay M, Abbasi A, Bergstrom EN et al. Visualizing and exploring patterns of large mutational events with SigProfilerMatrixGenerator. Bioinformatics; 2023 Feb. 10.1101/2023.02.03.527015. 10.1186/s12864-023-09584-y PMC10440861 37605126

[29] Zhu H, Uusküla-Reimand L, Isaev K, Wadi L, Alizada A, Shuai S (2020). Candidate Cancer driver mutations in Distal Regulatory Elements and Long-Range chromatin Interaction Networks. Mol Cell.

[30] Tate JG, Bamford S, Jubb HC, Sondka Z, Beare DM, Bindal N (2019). COSMIC: the catalogue of somatic mutations in Cancer. Nucleic Acids Res.

[31] Franchi A, Innocenti DRD, Palomba A, Miligi L, Paiar F, Franzese C (2014). Low prevalence of K-RAS, EGF-R and BRAF mutations in Sinonasal Adenocarcinomas. Implications for Anti-EGFR treatments. Pathol Oncol Res.

[32] Rooper LM, Thompson LDR, Gagan J, Hwang JSG, London NR, Mikula MW (2022). Low-grade non-intestinal-type sinonasal adenocarcinoma: a histologically distinctive but molecularly heterogeneous entity. Mod Pathol.

[33] Sánchez-Fernández P, Riobello C, Costales M, Vivanco B, Cabal VN, García-Marín R (2021). Next-generation sequencing for identification of actionable gene mutations in intestinal-type sinonasal adenocarcinoma. Sci Rep.

[34] Sjöstedt S, Schmidt AY, Vieira FG, Willemoe GL, Agander TK, Olsen C (2021). Major driver mutations are shared between sinonasal intestinal-type adenocarcinoma and the morphologically identical colorectal adenocarcinoma. J Cancer Res Clin Oncol.

[35] Kopanos C, Tsiolkas V, Kouris A, Chapple CE, Albarca Aguilera M, Meyer R et al. VarSome: the human genomic variant search engine. Wren J, editor. Bioinformatics. 2019;35:1978–80. 10.1093/bioinformatics/bty897 PMC6546127 30376034

[36] Stelzer G, Plaschkes I, Oz-Levi D, Alkelai A, Olender T, Zimmerman S (2016). VarElect: the phenotype-based variation prioritizer of the GeneCards suite. BMC Genomics.

[37] Leivo I, Holmila R, Luce D, Steiniche T, Dictor M, Heikkilä P (2021). Occurrence of Sinonasal Intestinal-Type Adenocarcinoma and Non-intestinal-type Adenocarcinoma in two countries with different patterns of Wood Dust exposure. Cancers.

[38] Feng L, Xia Y, Garcia GE, Hwang D, Wilson CB (1995). Involvement of reactive oxygen intermediates in cyclooxygenase-2 expression induced by interleukin-1, tumor necrosis factor-alpha, and lipopolysaccharide. J Clin Invest.

[39] Long H, Shi T, Borm PJ, Määttä J, Husgafvel-Pursiainen K, Savolainen K (2004). ROS-mediated TNF-α and MIP-2 gene expression in alveolar macrophages exposed to pine dust. Part Fibre Toxicol.

[40] Messerschmidt C, Obermayer B, Klinghammer K, Ochsenreither S, Treue D, Stenzinger A (2020). Distinct immune evasion in APOBEC -enriched, HPV ‐negative HNSCC. Int J Cancer.

[41] Perez-Ordonez B (2004). Expression of mismatch repair proteins, catenin, and E cadherin in intestinal-type sinonasal adenocarcinoma. J Clin Pathol.

[42] Martínez JG, Pérez-Escuredo J, López F, Suárez C, Álvarez‐Marcos C, Llorente JL (2009). Microsatellite instability analysis of sinonasal carcinomas. Otolaryngol Neck Surg.

[43] Ariza M, Luis Llorente J, Alvarez-Marcas C, Baragaño L, Salas A, Rodriguez Prado N (2004). Comparative genomic hybridization in primary sinonasal adenocarcinomas: sinonasal adenocarcinomas and CGH. Cancer.

[44] Korinth D, Pacyna-Gengelbach M, Deutschmann N, Hattenberger S, Bockmühl U, Dietel M (2005). Chromosomal imbalances in wood dust-related adenocarcinomas of the inner nose and their associations with pathological parameters. J Pathol.

[45] Lancho O, Herranz D (2018). The MYC Enhancer-ome: long-range transcriptional regulation of MYC in Cancer. Trends Cancer.

[46] Sun D, Yu F, Ma Y, Zhao R, Chen X, Zhu J (2013). MicroRNA-31 activates the RAS Pathway and functions as an oncogenic MicroRNA in human colorectal Cancer by repressing RAS p21 GTPase activating protein 1 (RASA1). J Biol Chem.

[47] Hieggelke L, Heydt C, Castiglione R, Rehker J, Merkelbach-Bruse S, Riobello C (2021). Mismatch Repair Deficiency and somatic mutations in human sinonasal tumors. Cancers.

[48] Rose M, Burgess JT, O’Byrne K, Richard DJ, Bolderson E (2020). PARP inhibitors: clinical relevance, mechanisms of Action and Tumor Resistance. Front Cell Dev Biol.

[49] Pérez-Escuredo J, Martínez JG, Vivanco B, Marcos CÁ, Suárez C, Llorente JL (2012). Wood dust–related mutational profile of TP53 in intestinal-type sinonasal adenocarcinoma. Hum Pathol.

[50] Bossi P, Perrone F, Miceli R, Cantù G, Mariani L, Orlandi E (2013). Tp53 status as guide for the management of ethmoid sinus intestinal-type adenocarcinoma. Oral Oncol.

[51] Projetti F, Durand K, Chaunavel A, Léobon S, Lacorre S, Caire F (2013). Epidermal growth factor receptor expression and KRAS and BRAF mutations: study of 39 sinonasal intestinal-type adenocarcinomas. Hum Pathol.

[52] García-Inclán C, López F, Pérez-Escuredo J, Cuesta-Albalad MP, Vivanco B, Centeno I (2012). EGFR status and KRAS/BRAF mutations in intestinal-type sinonasal adenocarcinomas. Cell Oncol.

[53] Hwang JA, Kim D, Chun S, Bae S, Song JS, Kim MY (2018). Genomic profiles of lung cancer associated with idiopathic pulmonary fibrosis. J Pathol.

[54] Manic G, Signore M, Sistigu A, Russo G, Corradi F, Siteni S (2018). CHK1-targeted therapy to deplete DNA replication-stressed, p53-deficient, hyperdiploid colorectal cancer stem cells. Gut.

[55] Sakuta K, Sasaki Y, Abe Y, Sato H, Shoji M, Yaoita T (2020). Somatic alterations and mutational burden are potential predictive factors for metachronous development of early gastric cancer. Sci Rep.

[56] Bornholdt J, Hansen J, Steiniche T, Dictor M, Antonsen A, Wolff H (2008). K-rasmutations in sinonasal cancers in relation to wood dust exposure. BMC Cancer.

