# Supplementary material for: Genome-wide somatic mutation analysis of sinonasal adenocarcinoma with and without wood dust exposure
Source: Genes Environ. 2024 May 6;46:12. doi: 10.1186/s41021-024-00306-8 (PMC11071320; doi:10.1186/s41021-024-00306-8)
Supplement: Supplementary file 1 — Additional file 1. Library preparation protocol. This additional file contains the final library protocol that was used to produce most of the libraries after the initial library production and sequencing tests. [file 41021_2024_306_MOESM1_ESM.pdf]

Genome-wide somatic mutation analysis of sinonasal adenocarcinoma with and without wood dust exposure

Additional file 1 – library preparation protocol

Lauri J. Sipilä [a,b,c], Riku Katainen [a,b,d], Mervi Aavikko [a,b,d], Janne Ravantti [a,b,e], Iikki Donner [f], Rainer Lehtonen [a,b], Ilmo Leivo [g,h], Henrik Wolff [i,j], Reetta Holmila [i], Kirsti Husgafvel-Pursiainen [i (retired)], Lauri A. Aaltonen[a,b,k,l]

Affiliations

[a] Department of Medical and Clinical Genetics, University of Helsinki, Biomedicum Helsinki, PO Box 63 (Haartmaninkatu 8), FI-00014, Helsinki, Finland

[b] Applied Tumor Genomics, Research Programs Unit, University of Helsinki, Biomedicum Helsinki, PO Box 63 (Haartmaninkatu 8), FI-00014, Helsinki, Finland

[c] Finnish Cancer Registry, Unioninkatu 22, 00130, Helsinki, Finland

[d] Institute for Molecular Medicine Finland (FIMM), HiLIFE, University of Helsinki, Helsinki, Finland

[e] Molecular and Integrative Biosciences Research Programme, Faculty of Biological and Environmental Sciences, University of Helsinki, FI-00014 Finland

[f] Organismal and Evolutionary Biology Research Programme, Faculty of Biological and Environmental Sciences, University of Helsinki, Viikinkaari 9, 00014 Helsinki, Finland

[g] Institute of Biomedicine, Pathology, University of Turku, Kiinamyllynkatu 10 D 5035, 20520 Turku, Finland

[h] Turku University Central Hospital, 20521 Turku, Finland

[i] Finnish Institute of Occupational Health, PB 40, 00251 Helsinki, Finland

[j] Department of Pathology, University of Helsinki, PB 20, 00014 Helsinki, Finland

[k] Department of Biosciences and Nutrition, Karolinska Institutet, 141 83 Huddinge, Sweden

[l] iCAN Digital Precision Cancer Medicine Flagship, University of Helsinki, 00290 Helsinki, Finland

The sequencing library protocol followed the standard KAPA library amplification kit protocol closely.

1. Input DNA: 600 ng phenol-chloroform-extracted sample DNA, diluted to 50 µl volume with (PCR-grade) water.
2. FFPE repair (NEBNext FFPE DNA repair mix, New England BioLabs, USA):
    2.1. Addition of 3.5 µl (PCR-grade) water, 6.5 µl FFPE repair buffer, 2 µl repair mix into the DNA dilution
    2.2. Incubation at 20 °C for 15 minutes.
    2.3. Cleanup with 186 µl AMPure XP beads (Beckman Coulter) following standard steps, final elution to 46 µl from which 44 µl is retained
3. S1 nuclease fragmentation
    3.1. Into product from 2.3., addition of 5 µl 10X Reaction Buffer (Promega) 0.3 µl S1 Nuclease (Promega) at 83 U/µl, 0.7 µl of (PCR grade) water.
    3.2. Incubation at 37 °C for 30 minutes.
    3.3. Termination with 2 µl 0.5 M EDTA.
    3.4. Incubation at 70 °C for 10 minutes.
    3.5. Cleanup with 80 µl AMPure XP beads following standard steps, final elution to 52 µl from which 50 µl is retained.
4. End repair, A-tailing
    4.1. Into product from 3.5., addition of 7 µl End repair & A-tailing buffer (Roche) and 3 µl End repair & A-tailing Enzyme (Roche)
    4.2. In a thermal cycler, incubation at 20 °C for 30 minutes, 65 °C for 30 minutes, hold at 10 °C indefinitely. Each reaction split in equal amounts in two separate tubes.
5. Adapter ligation

5.1. Into products from 4.2addition of 2.5 µl 15 µM adapter stock (SeqCap Adaptor Kit A, Roche), 2.5 µl (PCR-grade) water, 15 µl HyperPrep Ligation Buffer (Roche), 5 µl HyperPrep DNA Ligase Enzyme (Roche).

5.2. Incubation at 20 °C for two hours.

5.3. Cleanup with 44 µl AMPure XP beads following standard steps, final elution to 25 µl from which 20 µl is retained.

6. Amplification

6.1. Master mix according to KAPA HyperPrep protocol, ie. per reaction 25 µl KAPA HiFi HotStart ReadyMix (2X), 5µl KAPA Library Amplification Primer Mix (10X). Master mix added to 20 µl of the adapter-ligated library from step 5.

6.2. In a thermal cycler:

6.2.1. 98 °C for 45 seconds.

6.2.2. 8 cycles of 98 °C for 15 seconds, 60 °C for 30 seconds, 72 °C for 30 seconds.

6.2.3. 72 °C for one minute.

6.2.4. 10 °C indefinitely.

6.3. Cleanup with 50 µl AMPure XP beads following standard steps, final elution to 52 µl from which 50 µl is retained.

7. In samples with aberrant products in QC electropherogram: reconditioning PCR using amplification protocol at step 6, with the exception of using 1-3 cycles during step 6.2.2.
