# Supplementary material for: Genome-wide somatic mutation analysis of sinonasal adenocarcinoma with and without wood dust exposure
Source: Genes Environ. 2024 May 6;46:12. doi: 10.1186/s41021-024-00306-8 (PMC11071320; doi:10.1186/s41021-024-00306-8)
Supplement: Supplementary file 2 — Additional file 2. Supplementary figures and tables. This additional file contains mutation signature plots with hierarchical clusterings, BAF segment graphs, copy number call graphs and tables, quality control figures and tables, and supplementary alignment distance plots that visualize the effect of sequencing library preparation protocol on the number of chimeric reads. [file 41021_2024_306_MOESM2_ESM.pdf]

Genome-wide somatic mutation analysis of sinonasal adenocarcinoma with and without wood dust exposure

Additional file 2 – supplementary figures and tables

Lauri J. Sipilä [a,b,c], Riku Katainen [a,b,d], Mervi Aavikko [a,b,d], Janne Ravantti [a,b,e], Iikki Donner [f], Rainer Lehtonen [a,b], Ilmo Leivo [g,h], Henrik Wolff [i,j], Reetta Holmila [i], Kirsti Husgafvel-Pursiainen [i (retired)], Lauri A. Aaltonen[a,b,k,l]

Affiliations

[a] Department of Medical and Clinical Genetics, University of Helsinki, Biomedicum Helsinki, PO Box 63 (Haartmaninkatu 8), FI-00014, Helsinki, Finland

[b] Applied Tumor Genomics, Research Programs Unit, University of Helsinki, Biomedicum Helsinki, PO Box 63 (Haartmaninkatu 8), FI-00014, Helsinki, Finland

[c] Finnish Cancer Registry, Unioninkatu 22, 00130, Helsinki, Finland

[d] Institute for Molecular Medicine Finland (FIMM), HiLIFE, University of Helsinki, Helsinki, Finland

[e] Molecular and Integrative Biosciences Research Programme, Faculty of Biological and Environmental Sciences, University of Helsinki, FI-00014 Finland

[f] Organismal and Evolutionary Biology Research Programme, Faculty of Biological and Environmental Sciences, University of Helsinki, Viikinkaari 9, 00014 Helsinki, Finland

[g] Institute of Biomedicine, Pathology, University of Turku, Kiinamyllynkatu 10 D 5035, 20520 Turku, Finland

[h] Turku University Central Hospital, 20521 Turku, Finland

[i] Finnish Institute of Occupational Health, PB 40, 00251 Helsinki, Finland

[j] Department of Pathology, University of Helsinki, PB 20, 00014 Helsinki, Finland

[k] Department of Biosciences and Nutrition, Karolinska Institutet, 141 83 Huddinge, Sweden

[l] iCAN Digital Precision Cancer Medicine Flagship, University of Helsinki, 00290 Helsinki, Finland

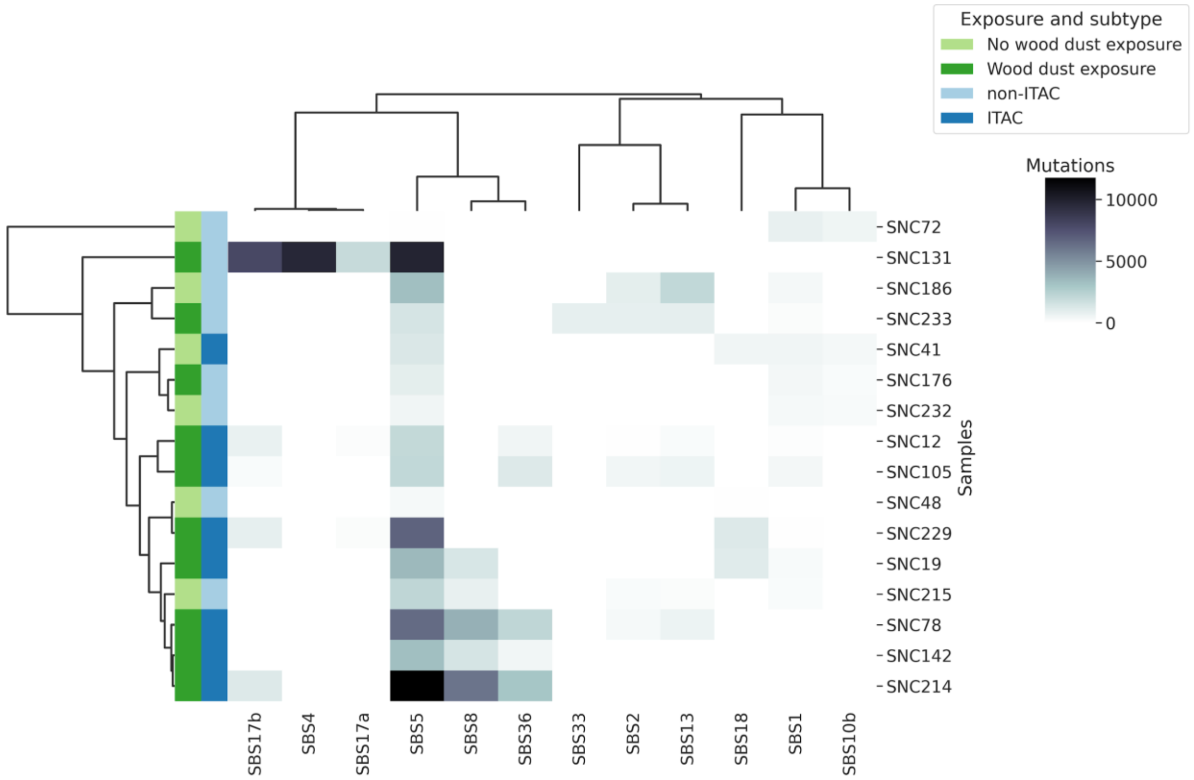

Figure S1. Single base substitution signatures of wood dust-exposed and non-exposed tumors produced with the SigProfiler method, with hierarchical clustering utilizing cosine distance and average linkage method. Input data is unscaled.

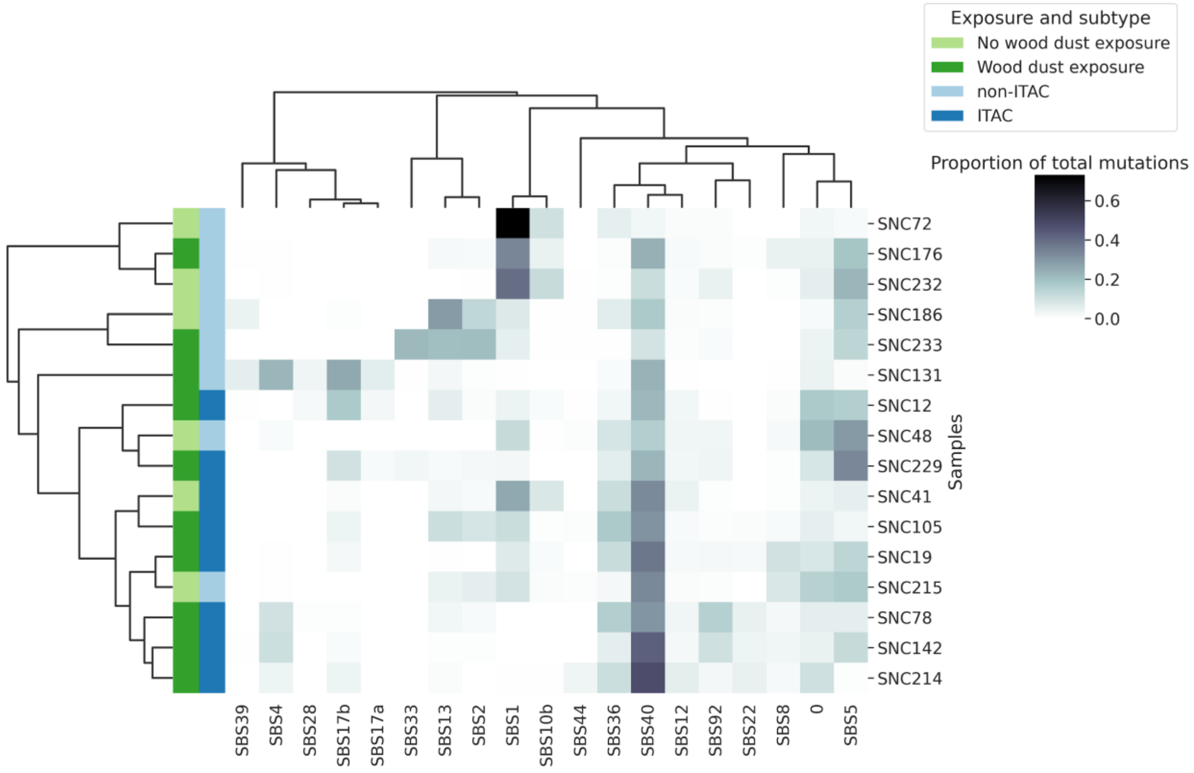

Figure S2. Single base substitution signatures of wood dust-exposed and non-exposed tumors produced with the HDP method, using SBS signature spectra as prior information, with hierarchical clustering utilizing cosine distance and average linkage method. Signature activities are scaled as the proportion of contribution to each sample's total mutation count. Component zero contains data unassigned to any other component during inference.

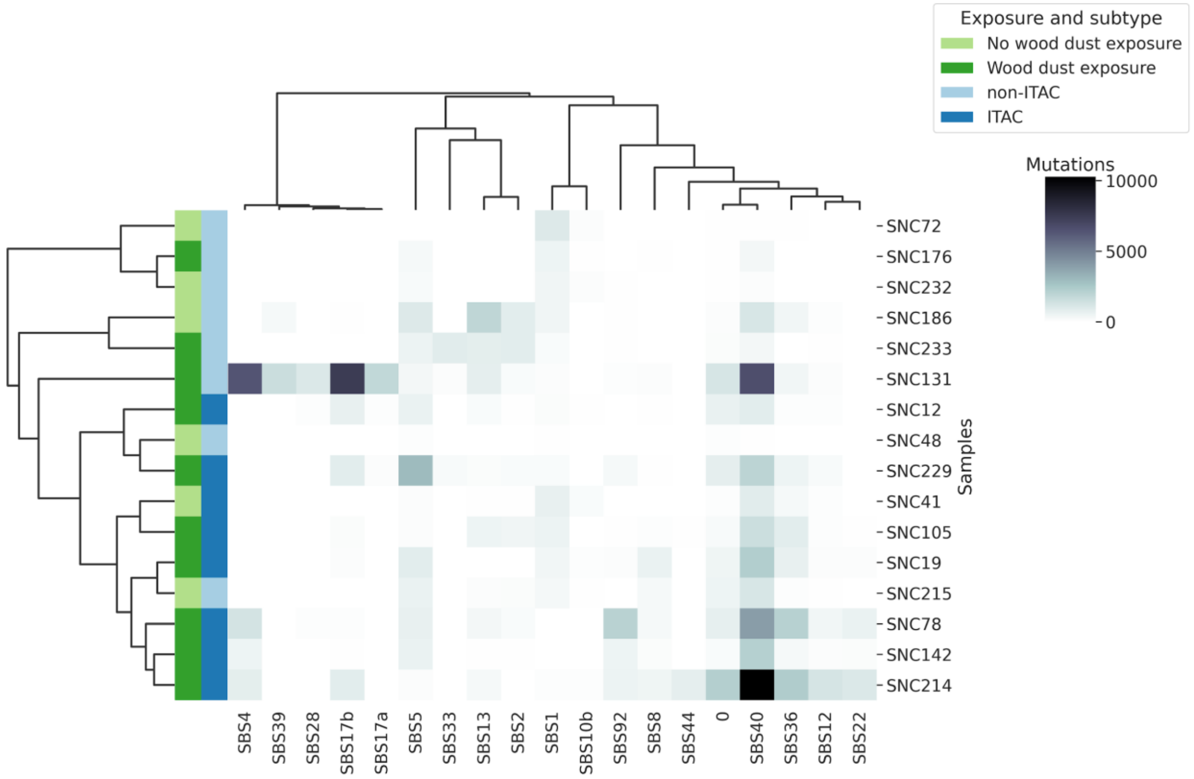

Figure S3. Single base substitution signatures of wood dust-exposed and non-exposed tumors produced with the HDP method, using SBS signature spectra as prior information, with hierarchical clustering utilizing cosine distance and average linkage method. Input data is unscaled. Component zero contains data unassigned to any other component during inference.

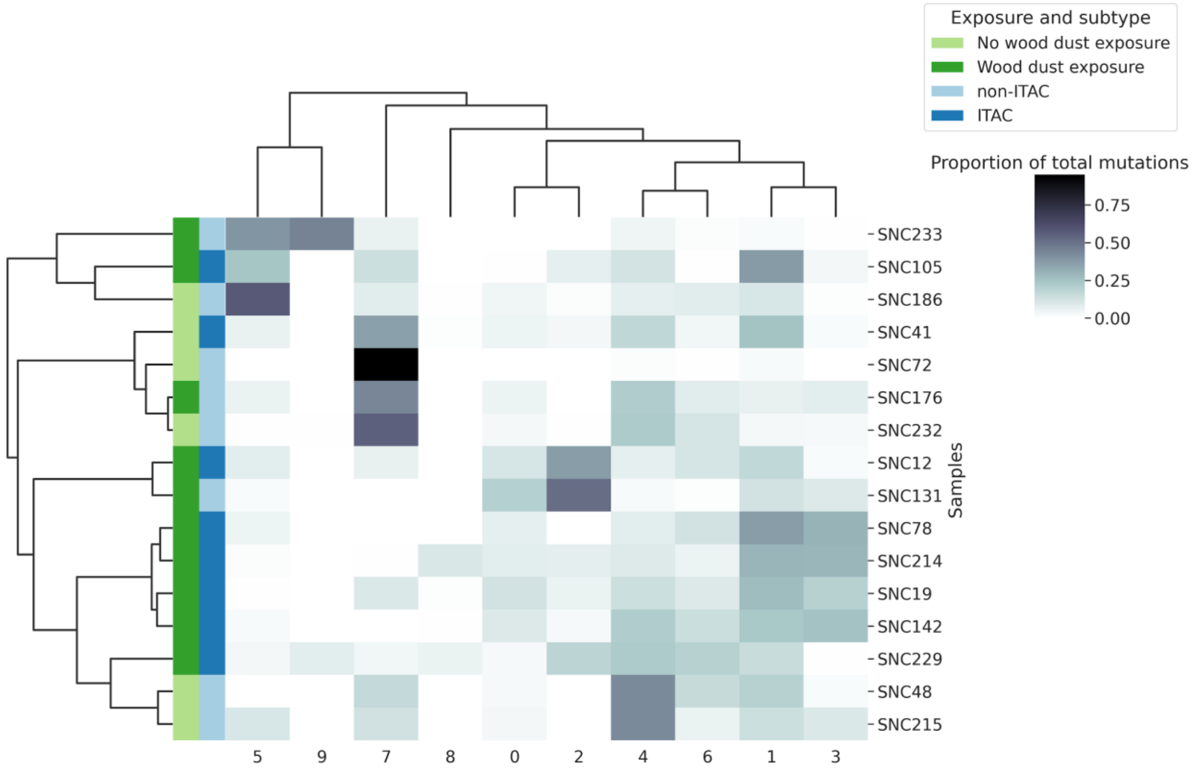

Figure S4. *De novo* mutational process components of wood dust-exposed and non-exposed tumors produced with the HDP method, without prior information and thus extracting *de novo* signatures, with hierarchical clustering utilizing cosine distance and average linkage method. Signature activities are scaled as the proportion of contribution to each sample's total mutation count.

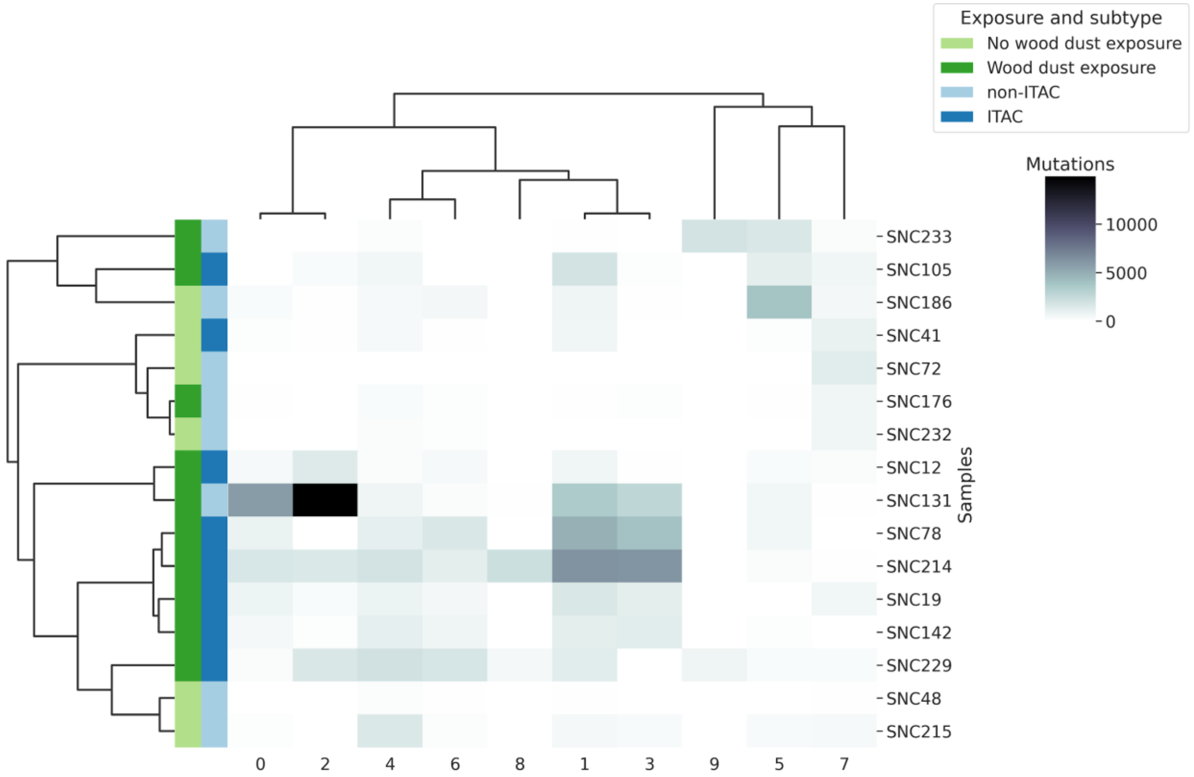

Figure S5. *De novo* mutational process components of wood dust-exposed and non-exposed tumors produced with the HDP method, without prior information and thus extracting *de novo* signatures, with hierarchical clustering utilizing cosine distance and average linkage method. Input data is unscaled.

Table S1. Cosine similarities of extracted *de novo* components and SBS signatures.

de novo component #	SBS signature	Cosine similarity
3	7c	0.90
1	3	0.78
9	3	0.76
1	40	0.73
1	39	0.73
4	39	0.72
4	3	0.70
1	89	0.69
7	21	0.69
9	40	0.69
9	39	0.68
5	3	0.67
9	5	0.66
1	5	0.64
5	40	0.62
7	3	0.62
4	40	0.62
9	89	0.61
4	89	0.61
2	34	0.60
1	8	0.60

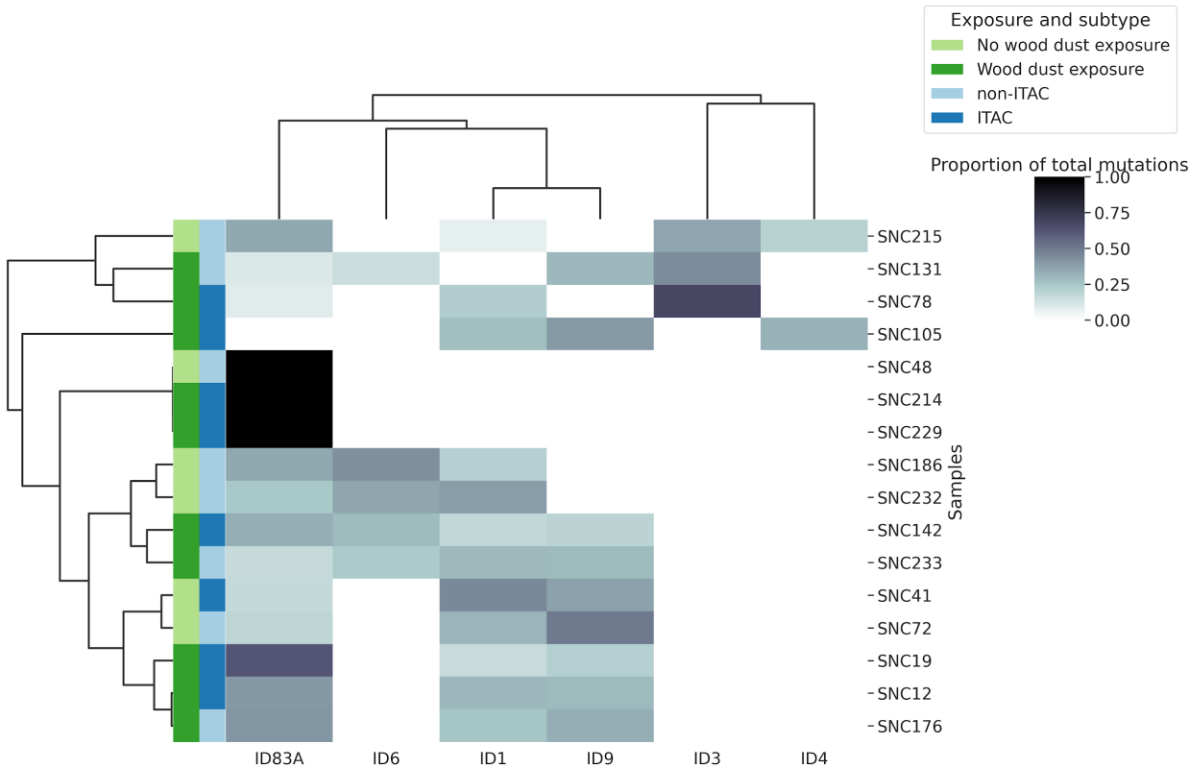

Figure S6. Small insertion and deletion mutational signatures of wood dust-exposed and non-exposed tumors produced with the SigProfiler method, with hierarchical clustering utilizing cosine distance and average linkage method. Signature activities are scaled as the proportion of contribution to each sample's total mutation count.

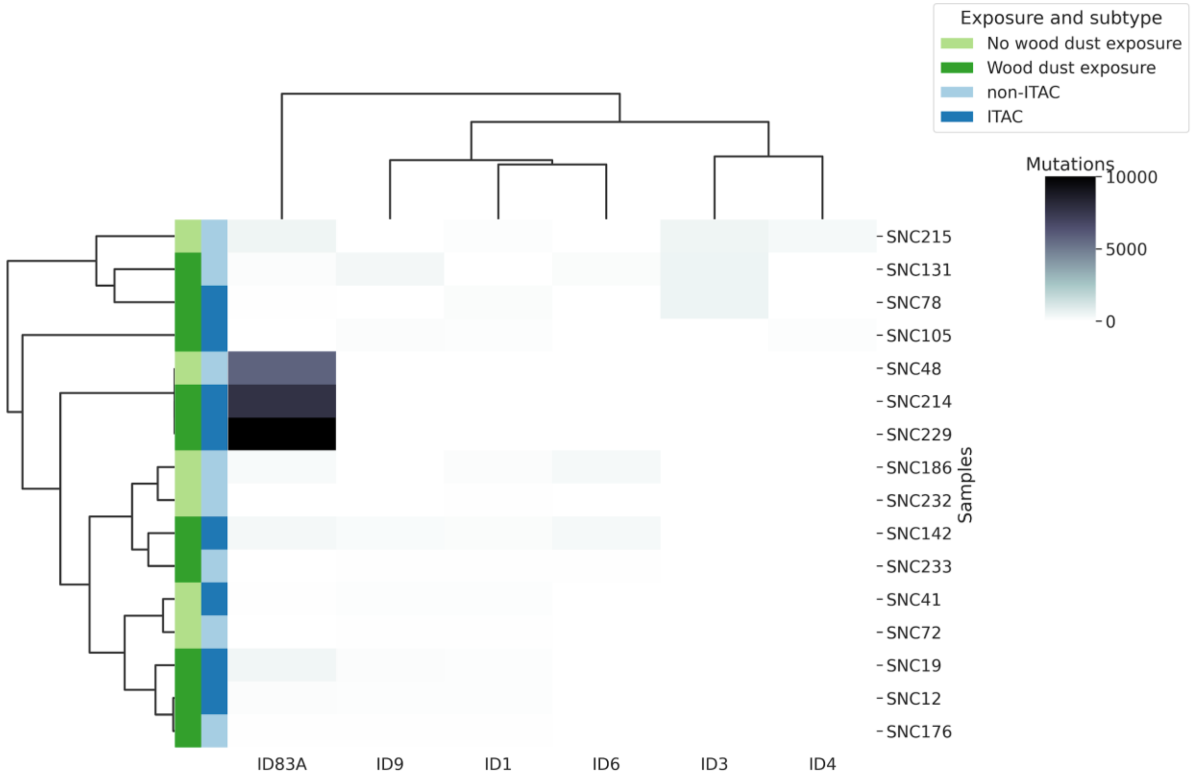

Figure S7. Small insertion and deletion mutational signatures of wood dust-exposed and non-exposed tumors produced with the SigProfiler method, with hierarchical clustering utilizing cosine distance and average linkage method. Input data is unscaled.

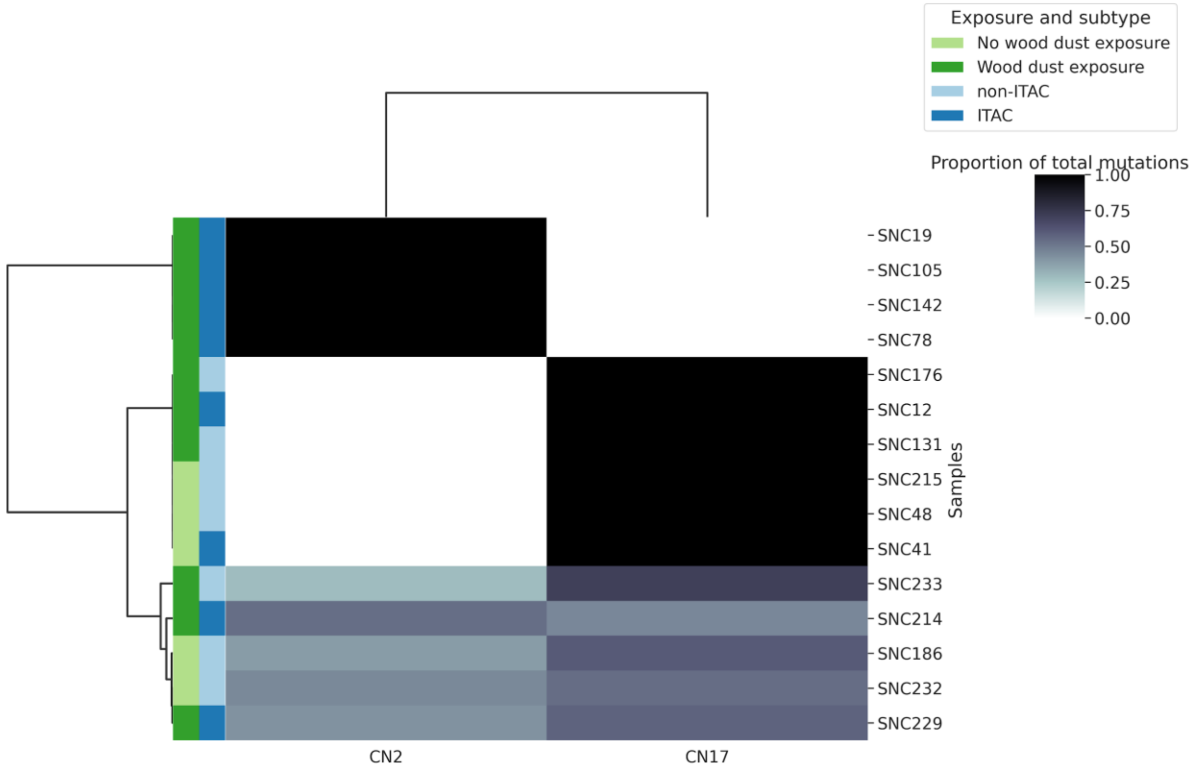

Figure S8. Copy number signatures of wood dust-exposed and non-exposed tumors produced with the SigProfiler method, with hierarchical clustering utilizing cosine distance and average linkage method. Signature activities are scaled as the proportion of contribution to each sample's total mutation count.

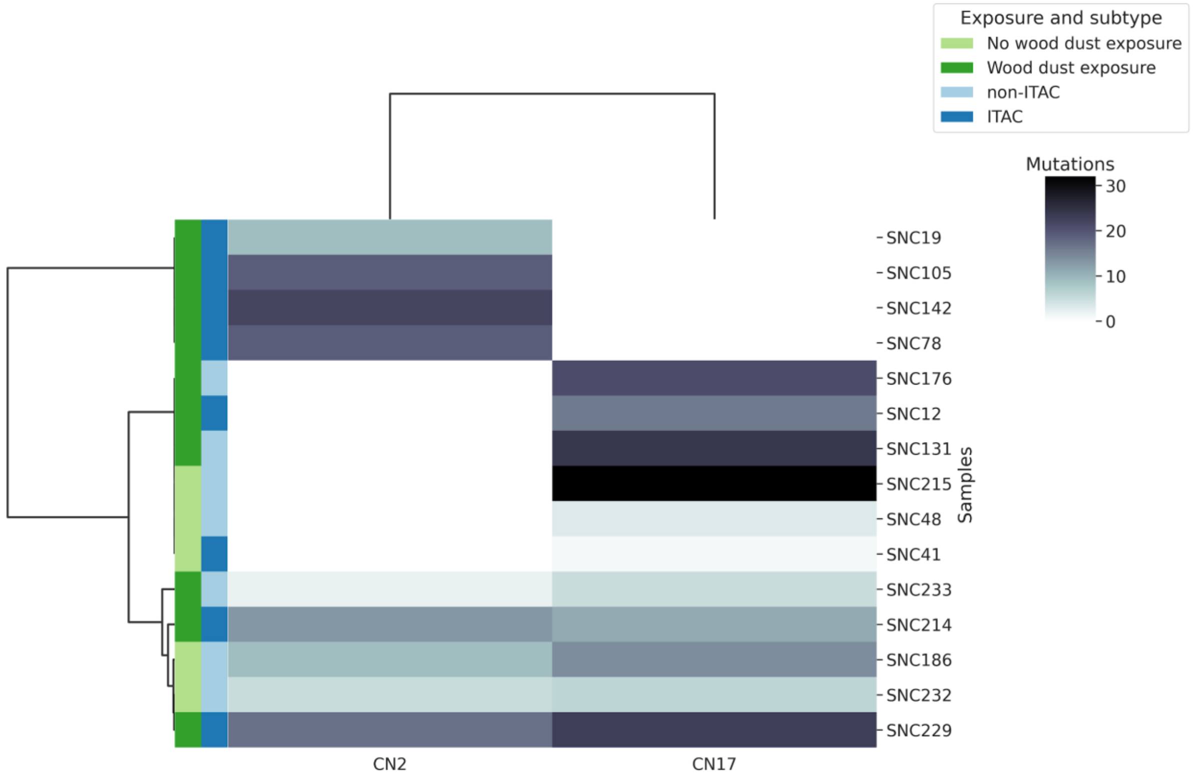

Figure S9. Copy number signatures of wood dust-exposed and non-exposed tumors produced with the SigProfiler method, with hierarchical clustering utilizing cosine distance and average linkage method. Input data is unscaled.

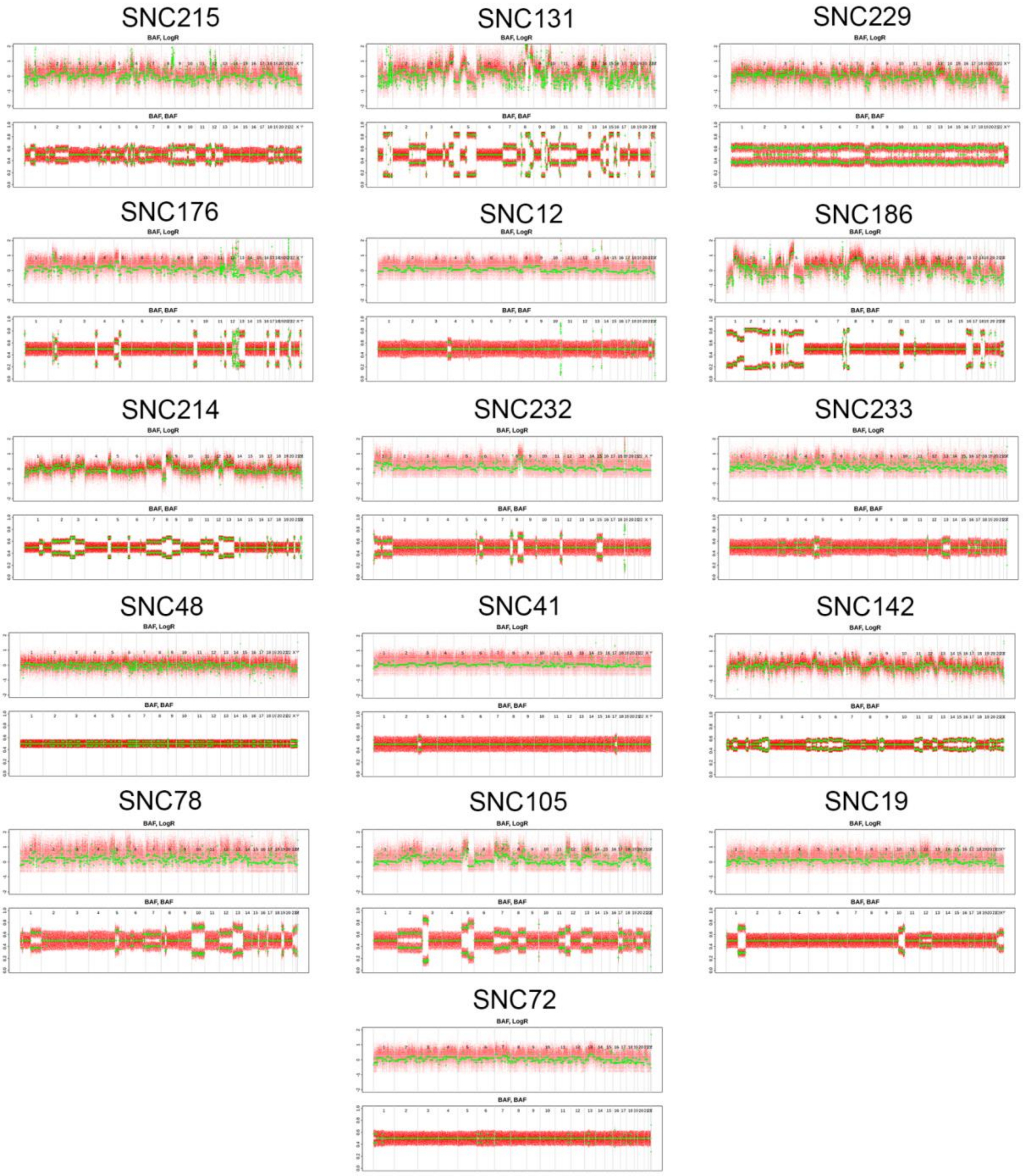

Figure S10. BAF segment graphs of each sample (ASCAT). Genomic loci sorted by chromosome number and position in ascending order on the X-axis. Y-axis contains log-transformed allele intensity ratios (sample-specific top graphs) and beta allele frequencies (sample-specific bottom graphs).

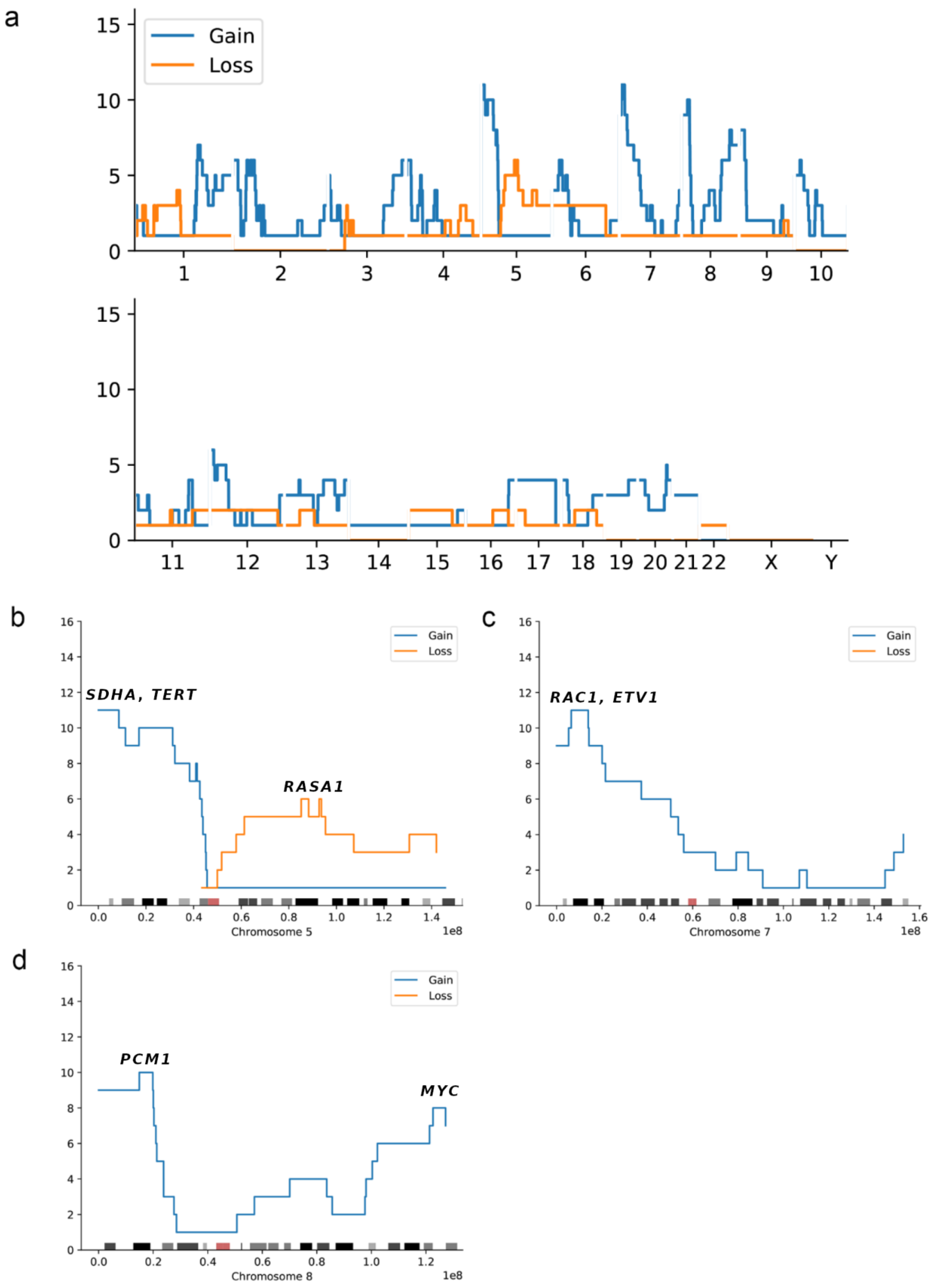

Figure S11. Copy number gains and losses in the sample set. **a)** Overview of the allelic imbalance in all chromosomes across all samples. **b)** Recurrent amplification in *SDHA* and

TERT, and loss of *RASA1* in chromosome 5. **c)** Recurrent amplification of *RAC1* and *ETV1* in chromosome 7. **d)** Recurrent amplification in *MYC* enhancer region in chromosome 8.

Table S2. Genes observed at or near CNV peaks and sample status for CNV at region.

Sample	Wood dust exposure	Subtype	TERT gain	RASA1 loss	RAC1 gain	PCM1 gain	MYC gain
SNC105	exposed	ITAC	x	x	x		x
SNC12	exposed	ITAC	x				
SNC142	exposed	ITAC	x	x	x		x
SNC19	exposed	ITAC			x	x	
SNC214	exposed	ITAC	x		x	x	x
SNC229	exposed	ITAC	x	x	x	x	x
SNC131	exposed	non-ITAC	x	x	x	x	x
SNC176	exposed	non-ITAC	x	x		x	
SNC233	exposed	non-ITAC	x			x	
SNC41	non-exposed	ITAC					
SNC78	non-exposed	ITAC	x		x	x	
SNC186	non-exposed	non-ITAC	x	x	x	x	x
SNC215	non-exposed	non-ITAC	x		x	x	
SNC232	non-exposed	non-ITAC			x		x
SNC48	non-exposed	non-ITAC				x	

SDHA and *ETV1* are not presented, as the information is redundant due to proximity to *TERT* and *RAC1*, respectively. Data for SNC72 is not presented, as it was used as the control sample in CNV calling.

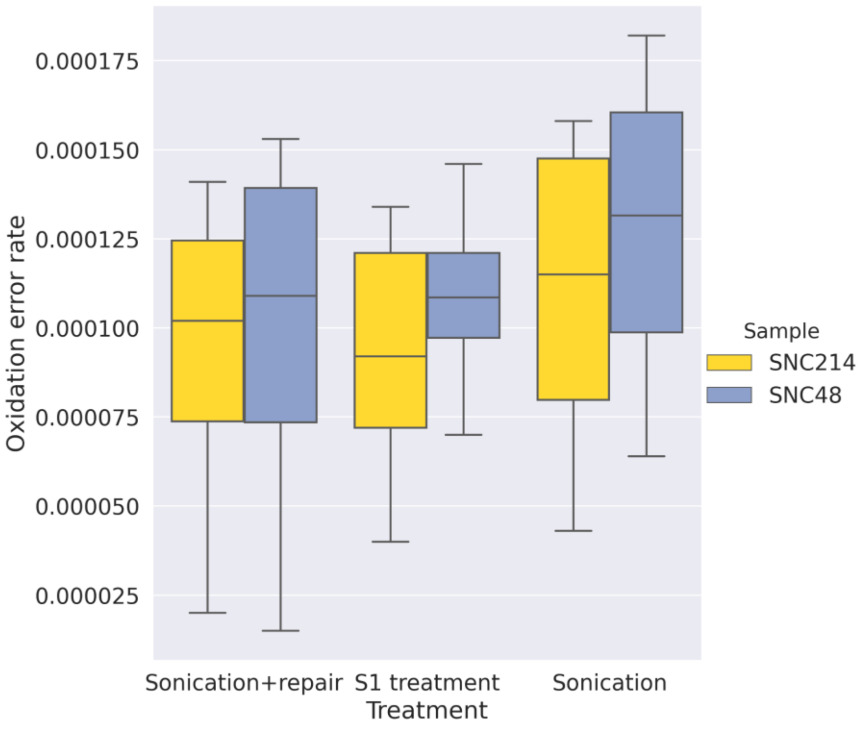

Figure S12. Oxidative artifact rates in all contexts, by sample and treatment. Error rate measured with Picard tool CollectOxoGMetrics. Sonication+repair includes the use of a commercial FFPE repair enzyme mix after DNA shearing with a Covaris sonicator.

Table S3. A subset of first library preparation test Picard alignment metrics.

Sample	Library type	Aligned base mismatches	Reads aligned in pairs	Chimeric reads	Strand balance
SNC214	Sonication and repair	0.0037	0.9991	0.0334	0.5011
SNC214	Covaris sonication	0.0039	0.9990	0.0447	0.5016
SNC214	S1 treatment	0.0039	0.9988	0.0321	0.5012
SNC48	Sonication and repair	0.0042	0.9988	0.1080	0.5058
SNC48	Covaris sonication	0.0033	0.9993	0.0672	0.5040
SNC48	S1 treatment	0.0035	0.9991	0.0856	0.5044

Metrics calculated after deduplication. Aggregated metrics for both read pairs. Aligned base mismatch rate: fraction of filter-passing and aligned bases mismatching the reference sequence. Reads aligned in pairs: fraction of reads with an aligned mate pair. Chimeric reads: fraction of reads where insert size exceeds 100kb or ends map to different chromosomes. Strand balance: fraction of reads mapped to positive strand vs. all reads.

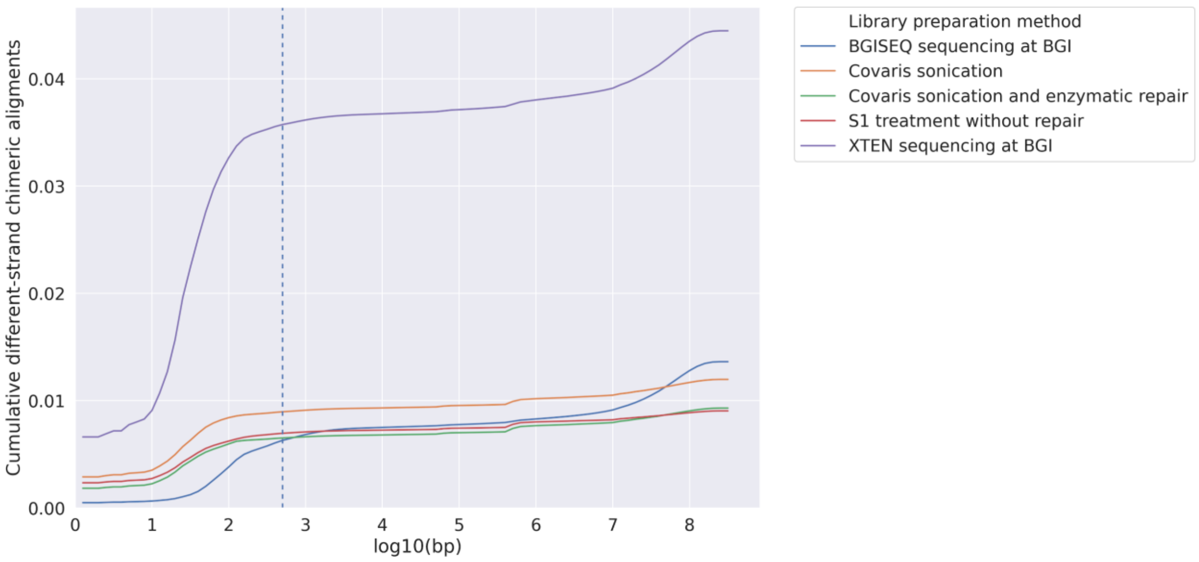

Figure S13. Sample SNC214 supplementary alignment distance plot. X-axis measures distance of same-chromosome-different-strand supplementary alignments of a read. Y-axis measures the fraction of same-chromosome-different-strand supplementary alignments in proportion to total alignment count. Vertical blue dashed line denotes 500 bp distance, based on the definition of SSAR. Different sequencing libraries of the sample presented, with line color denoting type of library preparation method.

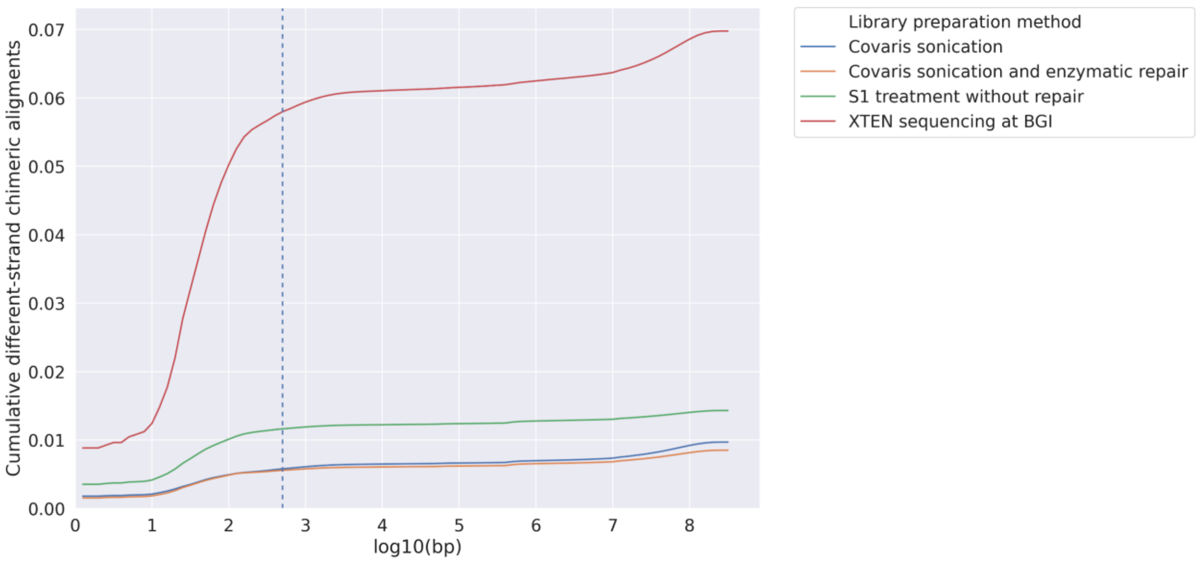

Figure S14. Sample SNC48 supplementary alignment distance plot. X-axis measures distance of same-chromosome-different-strand supplementary alignments of a read. Y-axis

measures the fraction of same-chromosome-different-strand supplementary alignments in proportion to total alignment count. Vertical blue dashed line denotes 500 bp distance, based on the definition of SSAR. Different sequencing libraries of the sample presented, with line color denoting type of library preparation method.

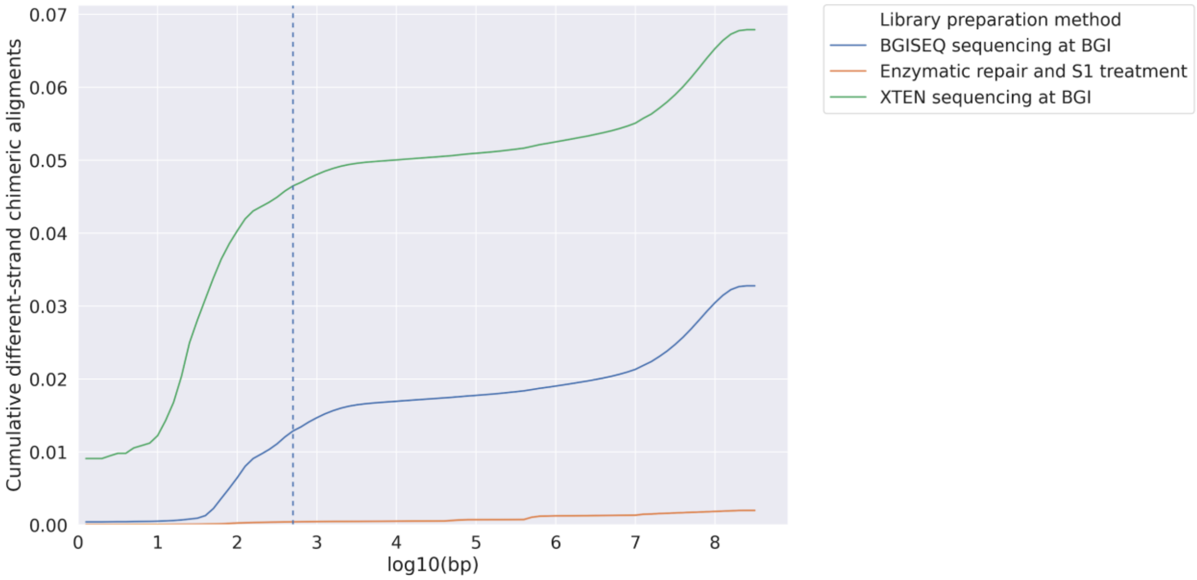

Figure S15. Sample SNC229 supplementary alignment distance plot. X-axis measures distance of same-chromosome-different-strand supplementary alignments of a read. Y-axis measures the fraction of same-chromosome-different-strand supplementary alignments in proportion to total alignment count. Vertical blue dashed line denotes 500 bp distance, based on the definition of SSAR. Different sequencing libraries of the sample presented, with line color denoting type of library preparation method.

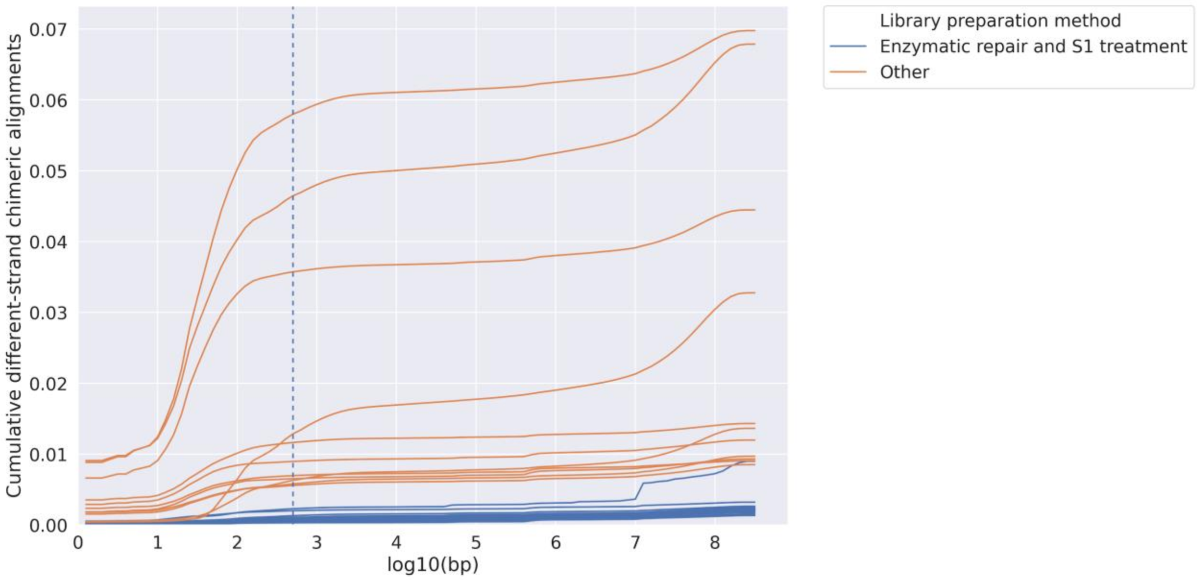

Figure S16. Complete supplementary alignment distance plot. X-axis measures distance of same-chromosome-different-strand supplementary alignments of a read. Y-axis measures the fraction of same-chromosome-different-strand supplementary alignments in proportion to total alignment count. Vertical blue dashed line denotes 500 bp distance, based on the definition of SSAR. All sequencing libraries presented from the sample set, with line color denoting type of library preparation method.

Table S4. Selected Picard alignment metrics of the complete sequencing library set.

Sample	Treatment	Replicate #	Total reads	Aligned base mismatches	Reads aligned in pairs	Chimeric reads	Strand balance
SNC176	Repair and S1	1	1.76E+09	0.0027	0.9996	0.0050	0.500
SNC186	Repair and S1	1	1.33E+09	0.0025	0.9998	0.0062	0.500
SNC78	Repair and S1	1	1.63E+09	0.0027	0.9995	0.0067	0.500
SNC131	Repair and S1	1	1.17E+09	0.0026	0.9996	0.0068	0.500
SNC142	Repair and S1	1	1.73E+09	0.0031	0.9995	0.0068	0.500
SNC215	Repair and S1	2	8.64E+08	0.0027	0.9992	0.0074	0.501

SNC186	Repair and S1	4	6.00E+08	0.0024	0.9994	0.0079	0.500
SNC142	Repair and S1	3	8.83E+08	0.0035	0.9992	0.0083	0.500
SNC19	Repair and S1	1	1.64E+09	0.0029	0.9993	0.0083	0.501
SNC131	Repair and S1	2	4.52E+08	0.0028	0.9992	0.0084	0.501
SNC105	Repair and S1	1	1.62E+09	0.0028	0.9992	0.0086	0.501
SNC215	Repair and S1	3	8.59E+08	0.0030	0.9991	0.0087	0.501
SNC232	Repair and S1	1	1.34E+09	0.0028	0.9997	0.0088	0.500
SNC12	Repair and S1	1	1.42E+09	0.0030	0.9997	0.0089	0.500
SNC131	Repair and S1	3	4.77E+08	0.0030	0.9990	0.0093	0.501
SNC186	Repair and S1	3	5.86E+08	0.0024	0.9994	0.0097	0.500
SNC41	Repair and S1	1	8.75E+08	0.0030	0.9992	0.0098	0.501
SNC229	Repair and S1	2	8.59E+08	0.0031	0.9992	0.0100	0.501
SNC233	Repair and S1	1	1.38E+09	0.0031	0.9996	0.0102	0.500
SNC142	Repair and S1	2	8.04E+08	0.0033	0.9993	0.0102	0.500
SNC186	Repair and S1	2	5.85E+08	0.0024	0.9992	0.0103	0.500
SNC229	Repair and S1	1	8.60E+08	0.0030	0.9991	0.0106	0.501
SNC72	Repair and S1	2	8.67E+08	0.0030	0.9991	0.0107	0.501
SNC214	S1 treatment	1	7.17E+08	0.0033	0.9988	0.0108	0.501
SNC131	Repair and S1	4	4.54E+08	0.0031	0.9987	0.0110	0.501
SNC131	Repair and S1	5	4.30E+08	0.0029	0.9988	0.0120	0.501
SNC72	Repair and	1	8.45E+08	0.0033	0.9990	0.0122	0.501

	S1						
SNC41	Repair and S1	2	7.84E+08	0.0030	0.9989	0.0131	0.501
SNC48	S1 treatment	1	7.33E+08	0.0028	0.9993	0.0141	0.501
SNC214	Sonication and repair	1	6.24E+08	0.0032	0.9991	0.0141	0.500
SNC214	Covaris sonication	1	6.54E+08	0.0034	0.9990	0.0150	0.501
SNC48	Sonication and repair	1	7.58E+08	0.0031	0.9991	0.0151	0.500
SNC48	Covaris sonication	1	7.10E+08	0.0034	0.9988	0.0194	0.500
SNC215	Repair and S1	1	1.04E+09	0.0030	0.9984	0.0370	0.503
SNC214	BGISEQ	1	9.24E+08	0.0042	0.9993	0.0370	0.500
SNC214	X Ten	1	9.39E+08	0.0049	0.9974	0.0442	0.500
SNC48	X Ten	1	9.96E+08	0.0044	0.9972	0.0565	0.500
SNC229	BGISEQ	1	8.91E+08	0.0044	0.9989	0.0731	0.500
SNC229	X Ten	1	9.55E+08	0.0053	0.9970	0.0757	0.500

Aggregated metrics for both read pairs presented. Amount of data generated varies between libraries, and duplicates have been removed. Table sorted by chimeric read rate. Replicate number: additional identifier separating different libraries produced from the same sample using the same library preparation method. Total reads: total amount of reads generated from the library. Aligned base mismatch rate: fraction of filter-passing and aligned bases mismatching the reference sequence. Reads aligned in pairs: fraction of reads with an aligned mate pair. Chimeric reads: fraction of reads where insert size exceeds 100kb or ends map to different chromosomes. Strand balance: fraction of reads mapped to positive strand vs. all reads.
